# Supplementary figures and images for: FBXO24 deletion causes abnormal accumulation of membraneless electron-dense granules in sperm flagella and male infertility
Source: eLife. 2024 Aug 20;13:RP92794. doi: 10.7554/eLife.92794 (PMC11335345; doi:10.7554/eLife.92794)

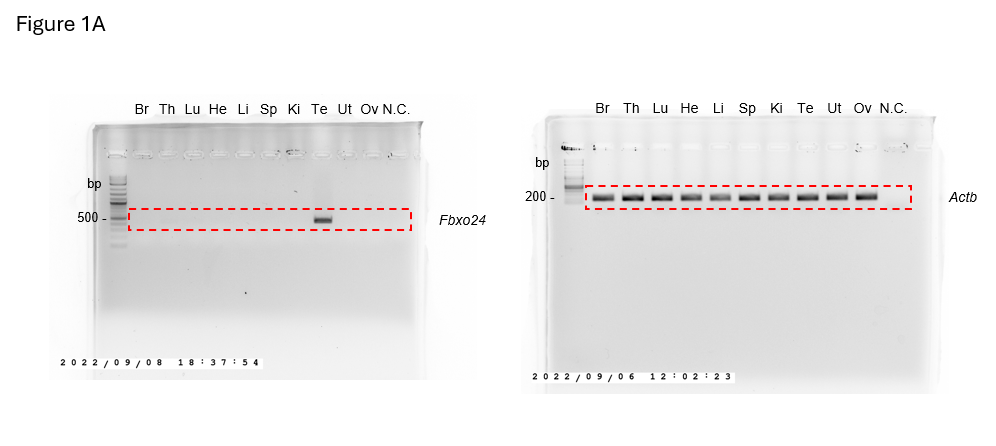

Supplement: Figure 1—source data 1. [file elife-92794-fig1-data1.zip › Figure 1-source data 1/Figure 1A_edited.tif]

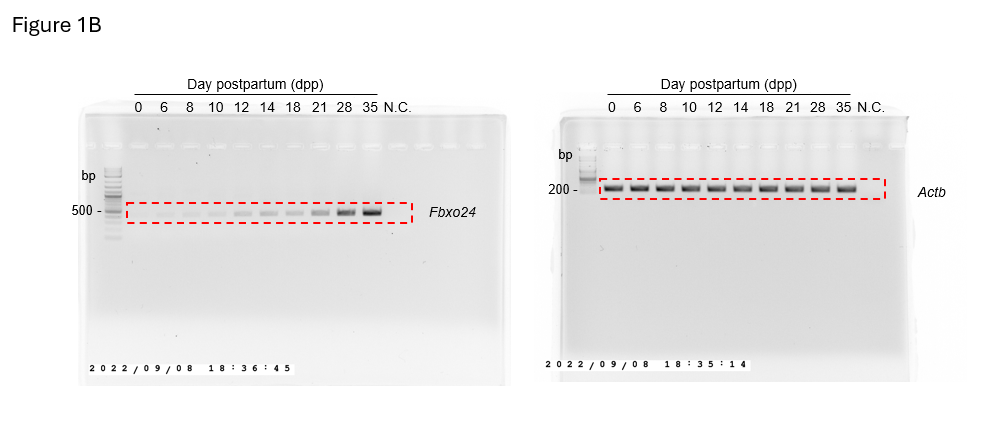

Supplement: Figure 1—source data 1. [file elife-92794-fig1-data1.zip › Figure 1-source data 1/Figure 1B_edited.tif]

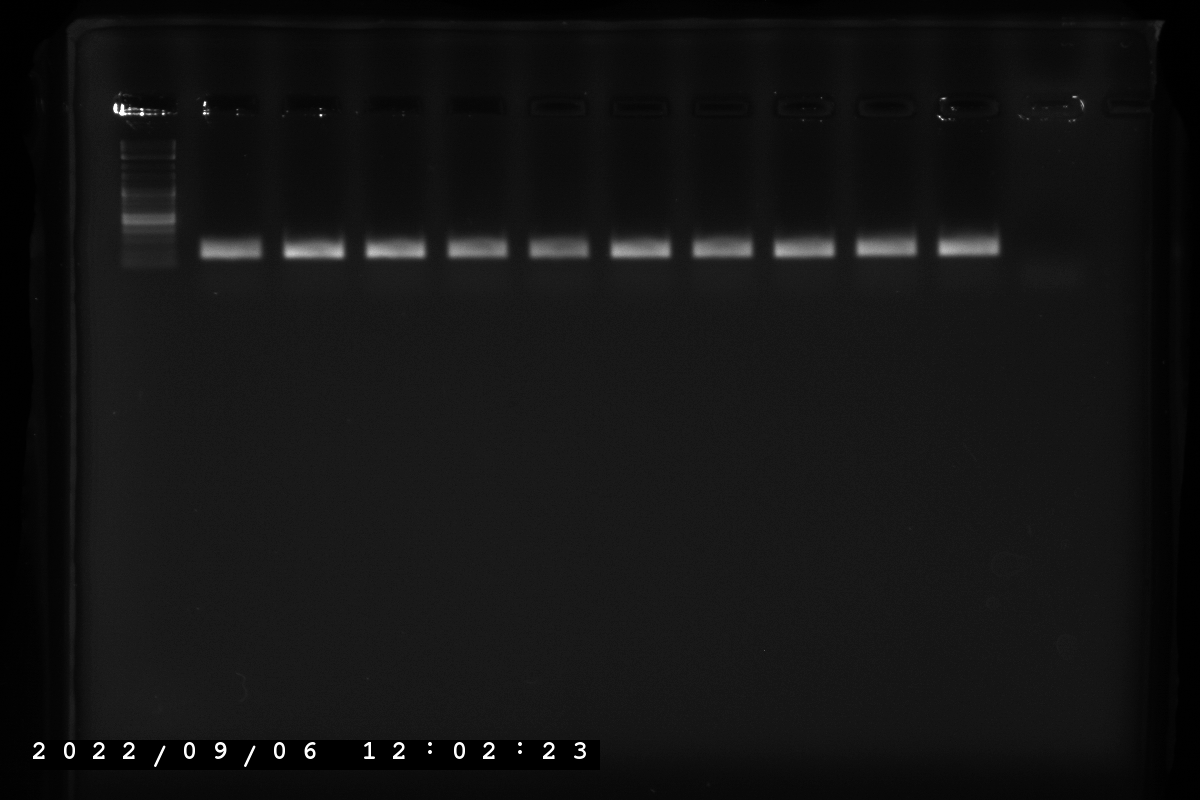

Supplement: Figure 1—source data 2. [file elife-92794-fig1-data2.zip › Figure 1-source data 2/Raw RT-PCR gel for Figure 1A_Actb_unedited.tiff]

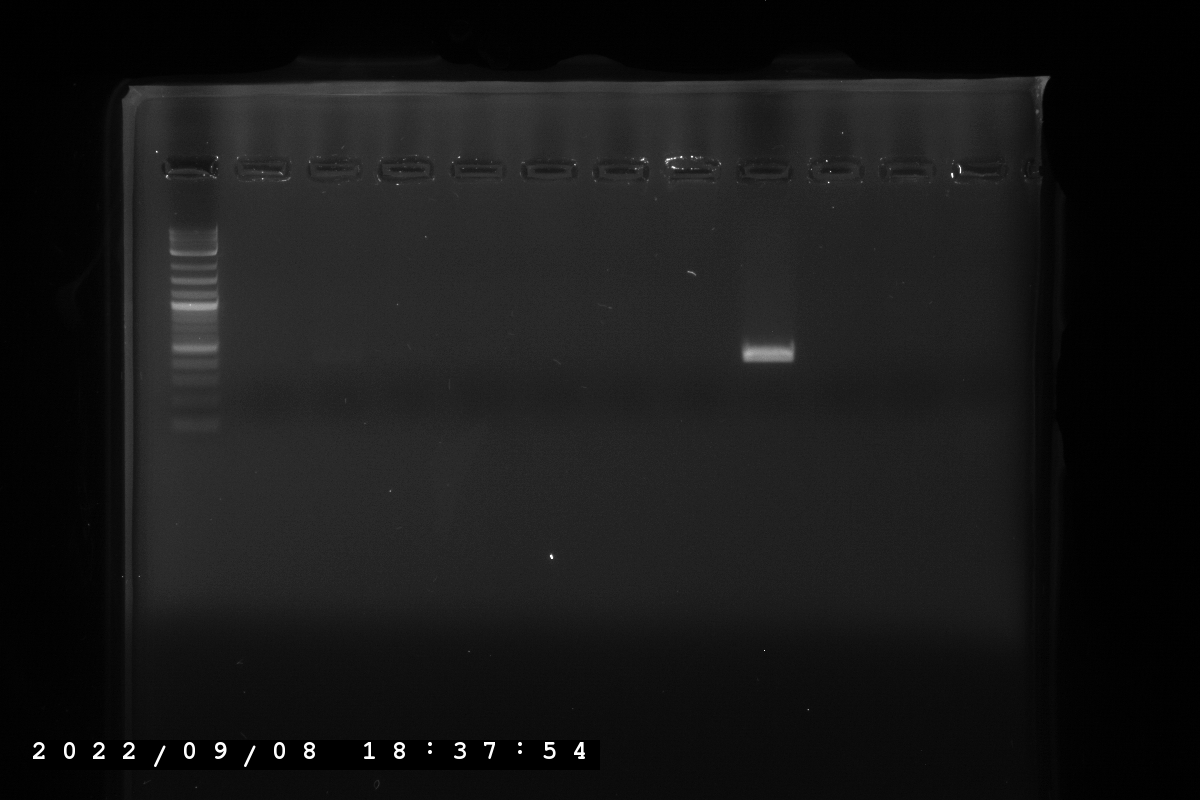

Supplement: Figure 1—source data 2. [file elife-92794-fig1-data2.zip › Figure 1-source data 2/Raw RT-PCR gel for Figure 1A_Fbxo24_unedited.tiff]

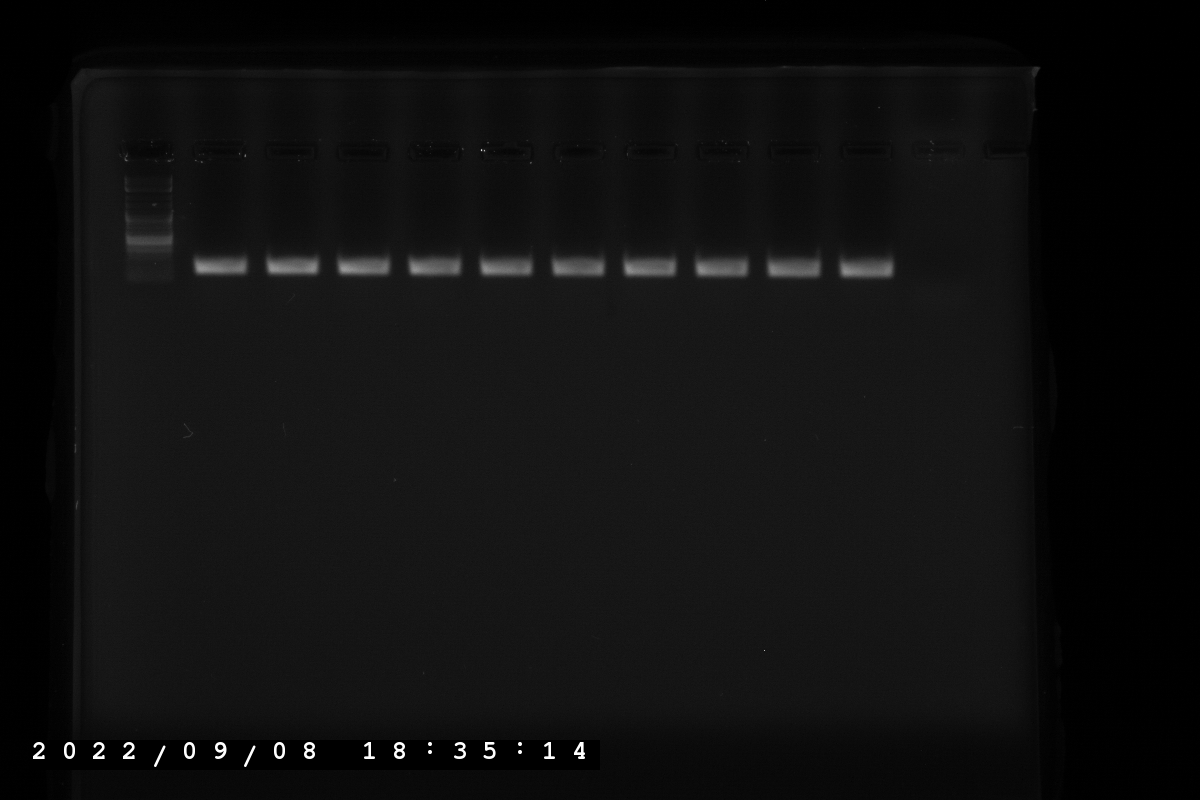

Supplement: Figure 1—source data 2. [file elife-92794-fig1-data2.zip › Figure 1-source data 2/Raw RT-PCR gel for Figure 1B_Actb_unedited.tiff]

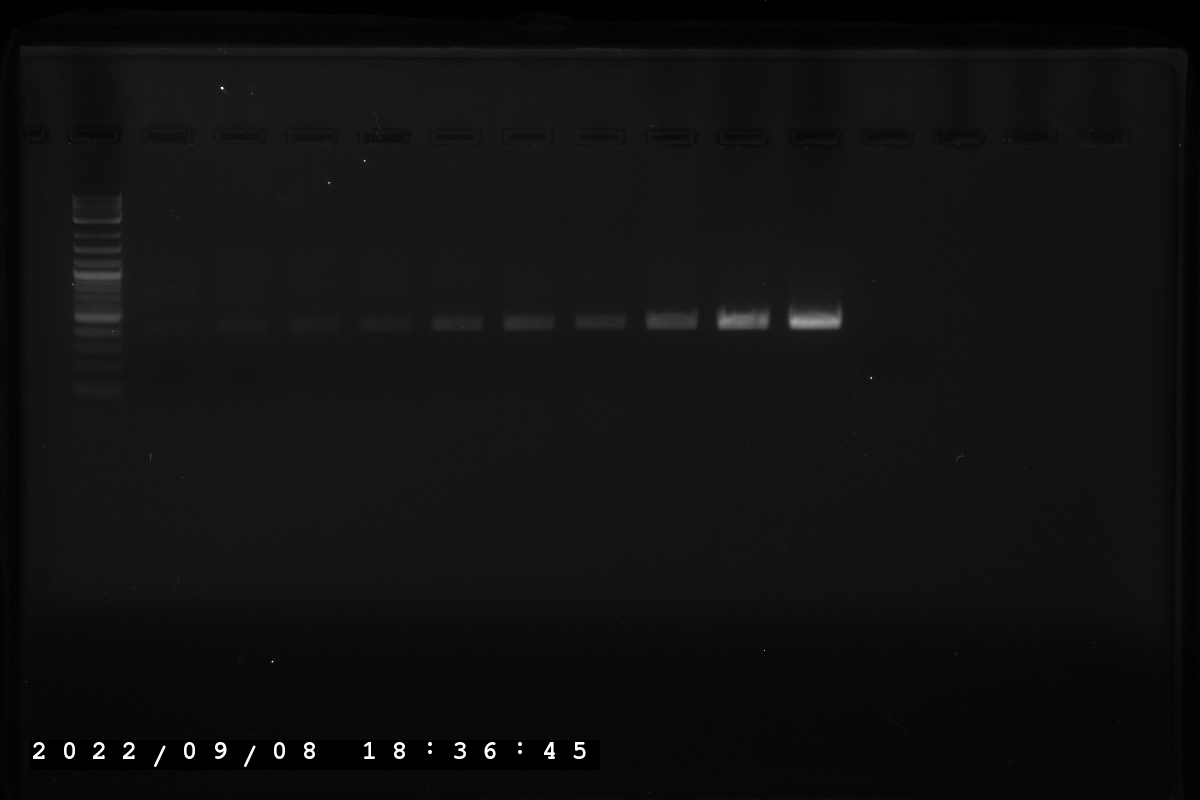

Supplement: Figure 1—source data 2. [file elife-92794-fig1-data2.zip › Figure 1-source data 2/Raw RT-PCR gel for Figure 1B_Fbxo24_unedited.tiff]

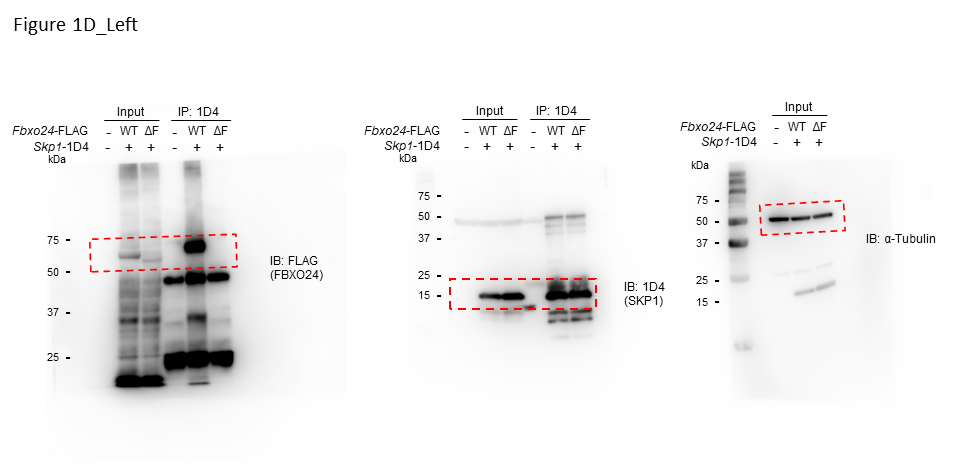

Supplement: Figure 1—source data 3. [file elife-92794-fig1-data3.zip › Figure 1-source data 3/Figure 1D_Left_edited.tif]

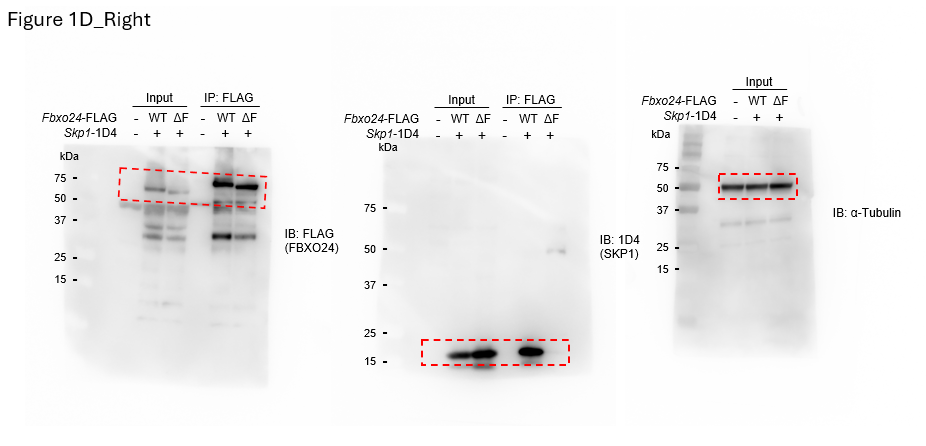

Supplement: Figure 1—source data 3. [file elife-92794-fig1-data3.zip › Figure 1-source data 3/Figure 1D_Right_edited.tif]

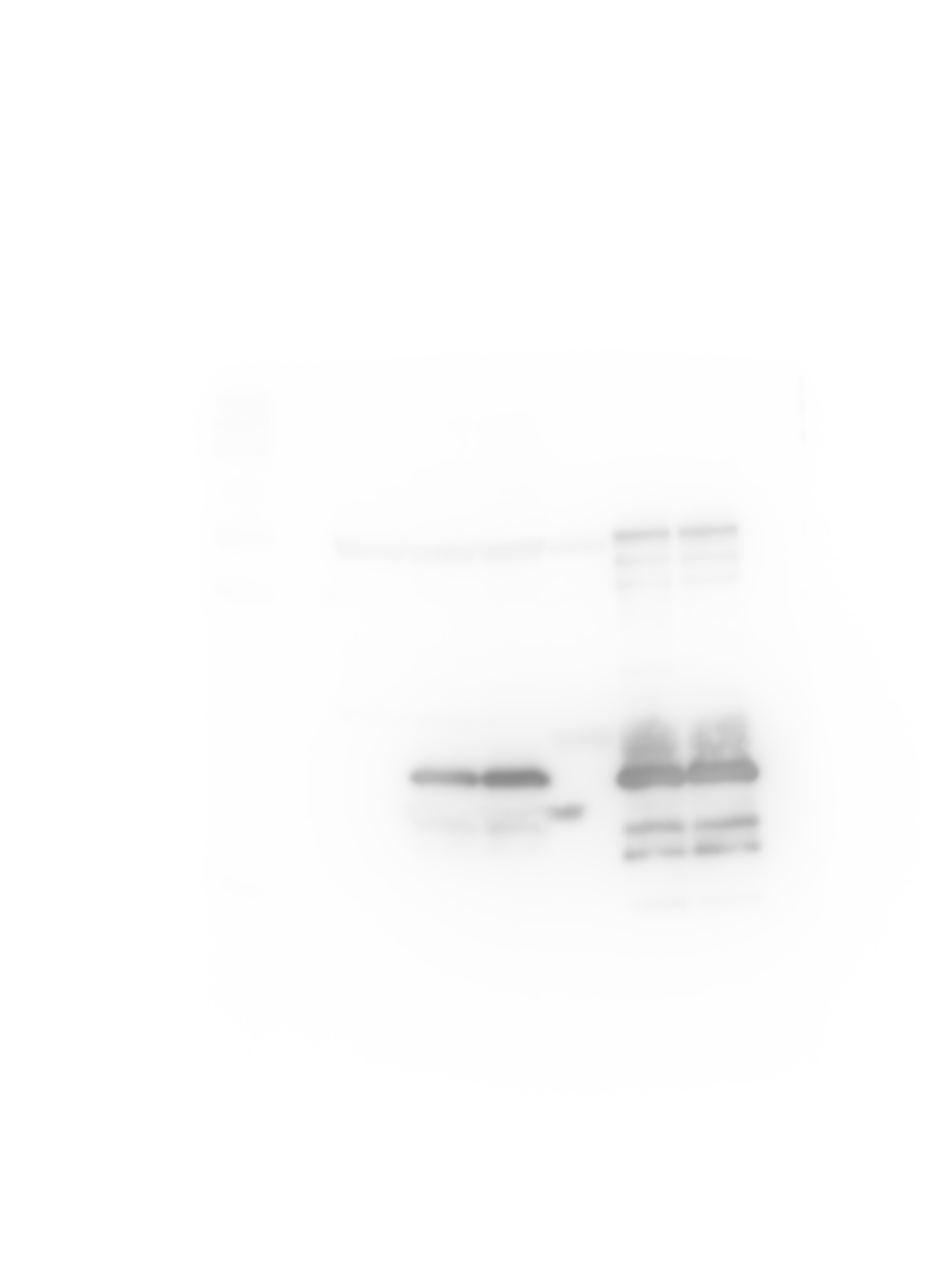

Supplement: Figure 1—source data 4. [file elife-92794-fig1-data4.zip › Figure 1-source data 4/Raw Western blot for Figure 1D_Left_1D4_unedited.tif]

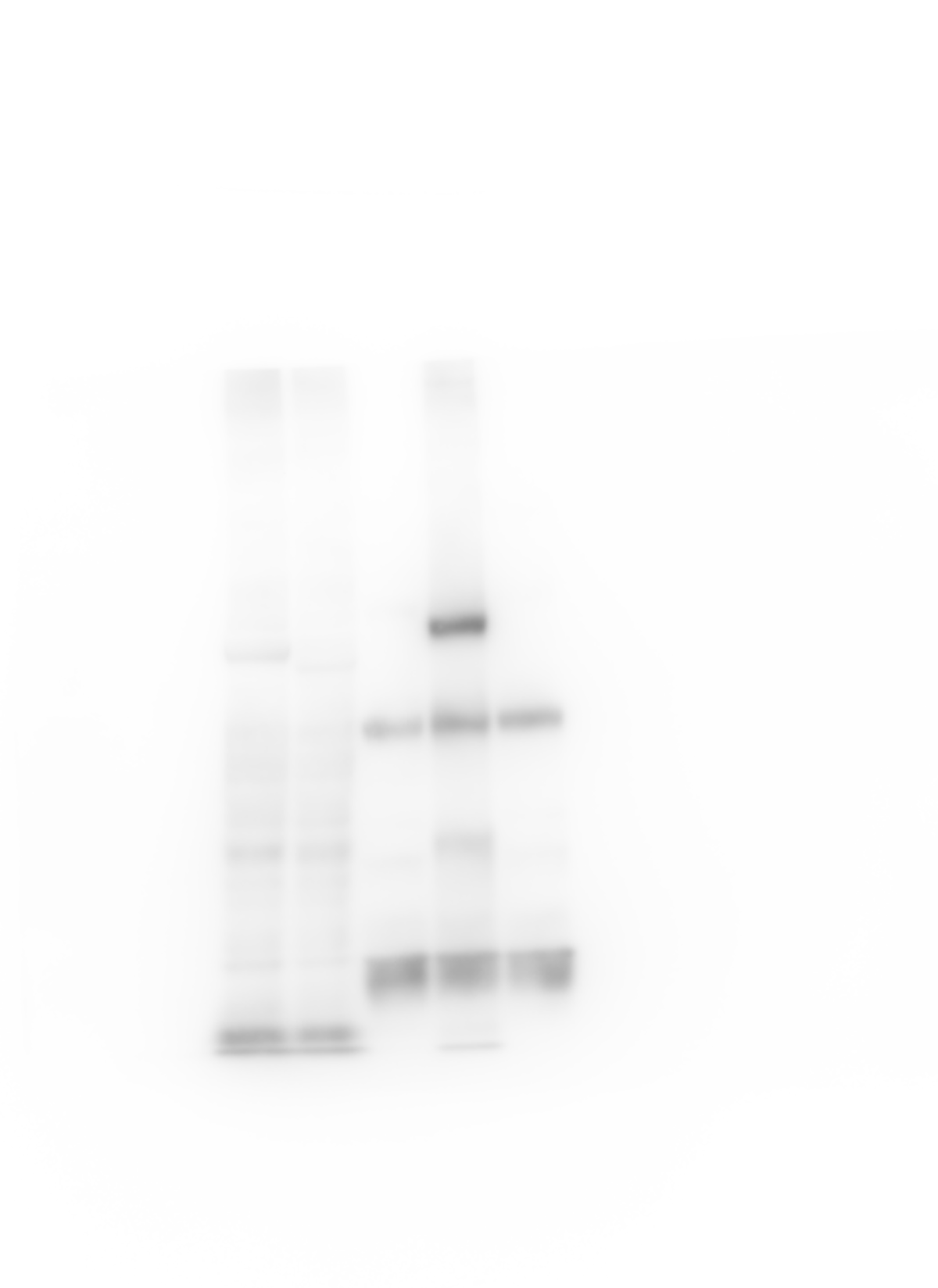

Supplement: Figure 1—source data 4. [file elife-92794-fig1-data4.zip › Figure 1-source data 4/Raw Western blot for Figure 1D_Left_FLAG_unedited.tif]

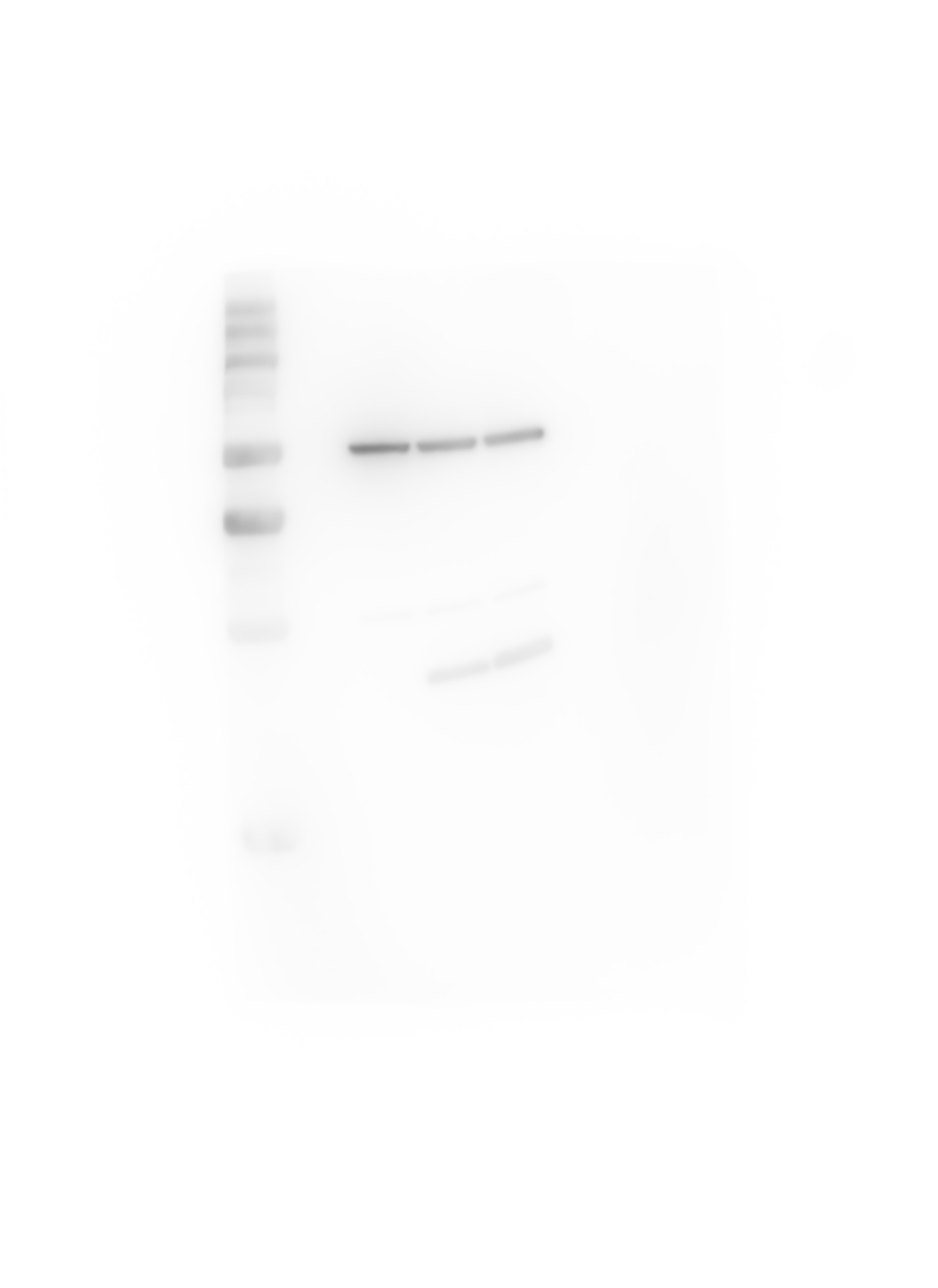

Supplement: Figure 1—source data 4. [file elife-92794-fig1-data4.zip › Figure 1-source data 4/Raw Western blot for Figure 1D_Left_â┐-Tubulin_unedited.tif]

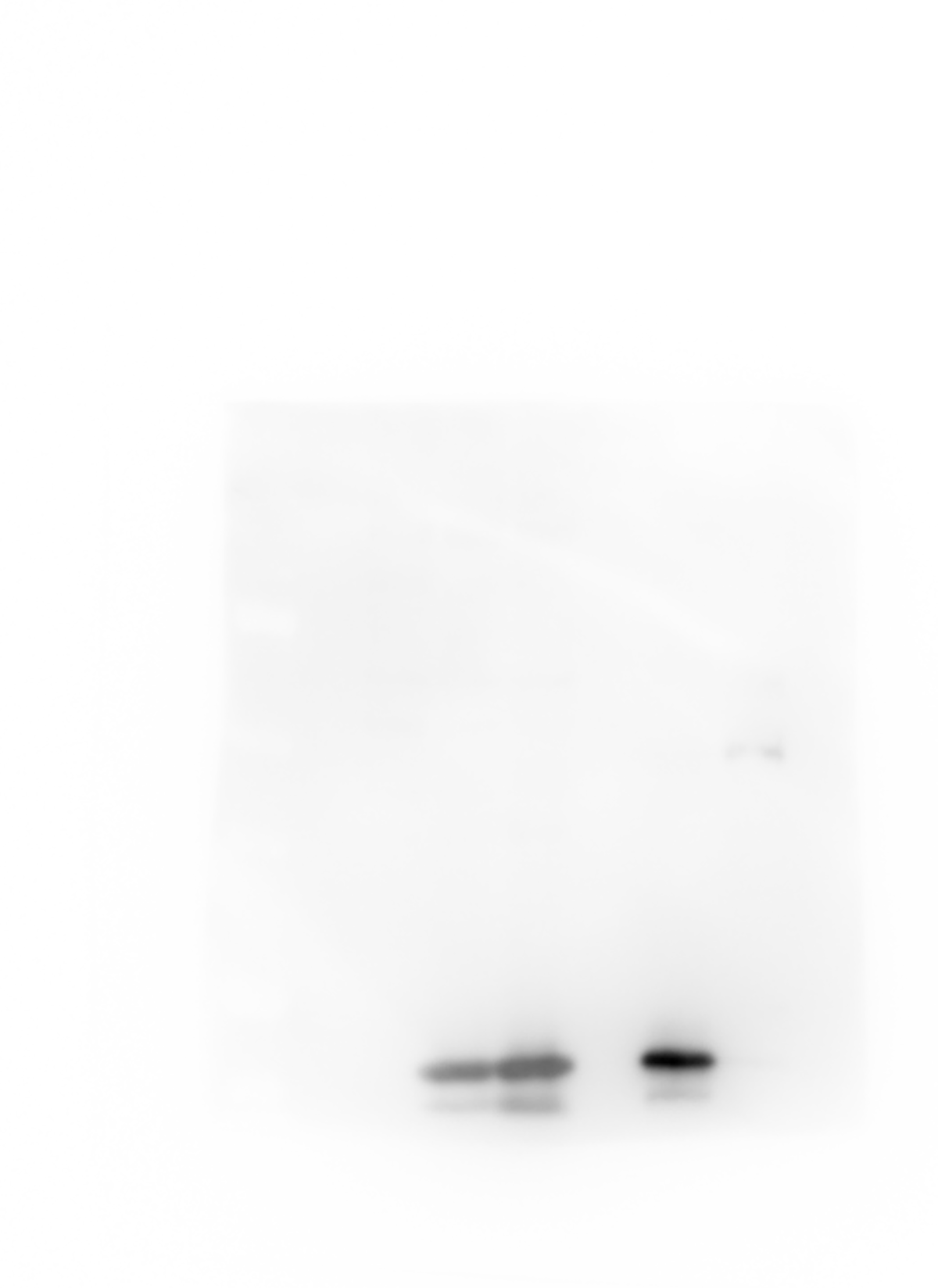

Supplement: Figure 1—source data 4. [file elife-92794-fig1-data4.zip › Figure 1-source data 4/Raw Western blot for Figure 1D_Right_1D4_unedited.tif]

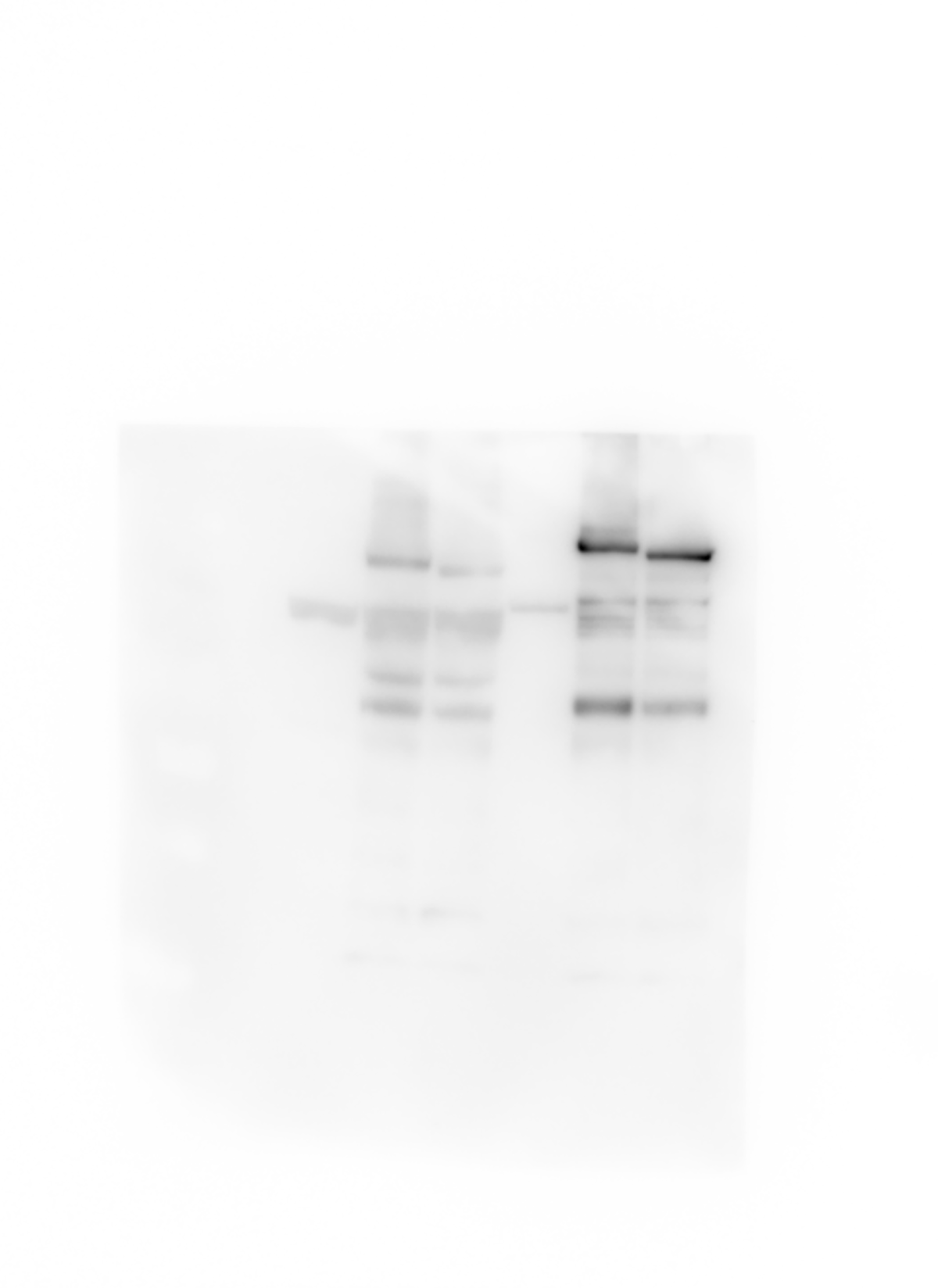

Supplement: Figure 1—source data 4. [file elife-92794-fig1-data4.zip › Figure 1-source data 4/Raw Western blot for Figure 1D_Right_FLAG_unedited.tif]

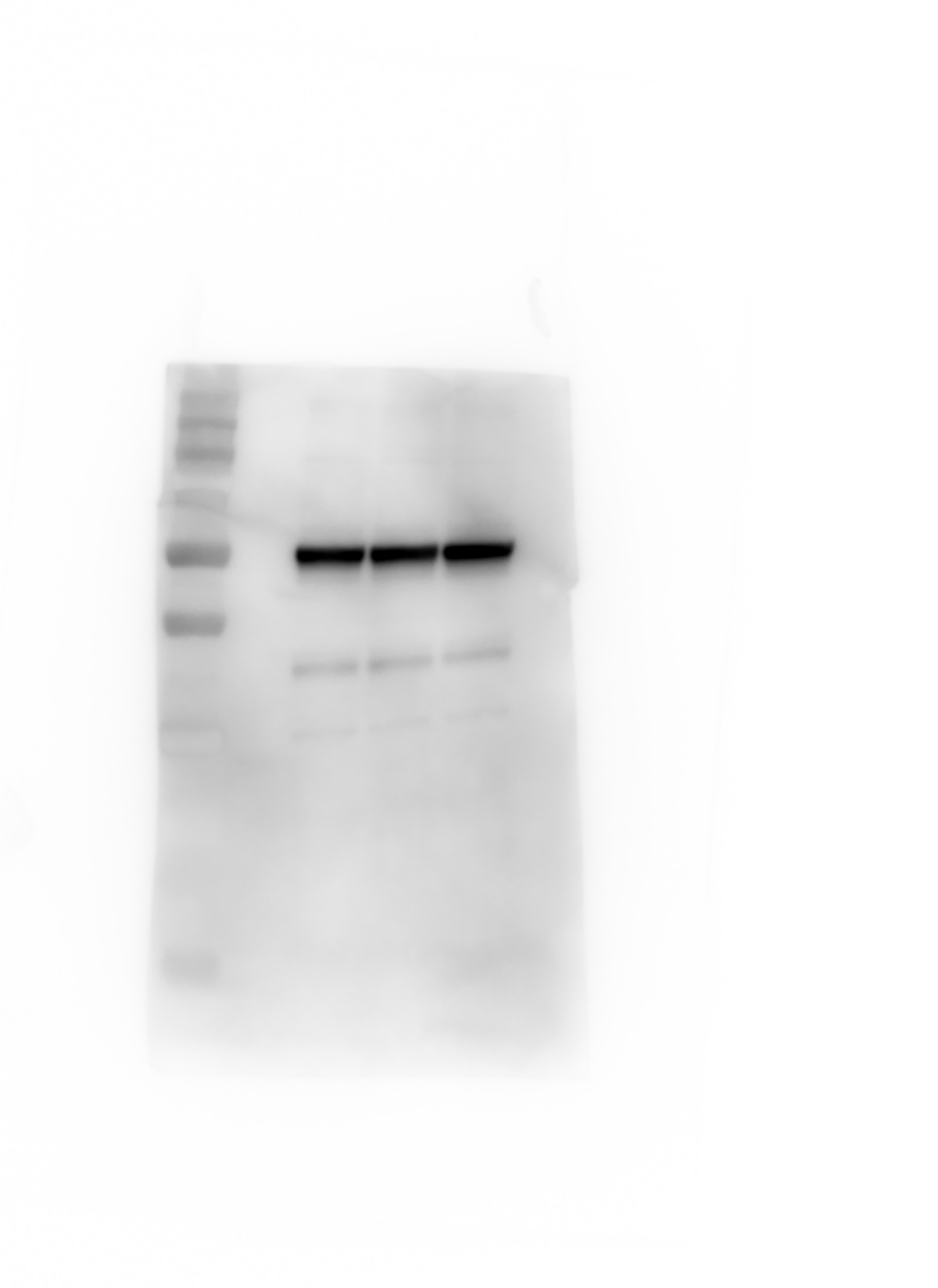

Supplement: Figure 1—source data 4. [file elife-92794-fig1-data4.zip › Figure 1-source data 4/Raw Western blot for Figure 1D_Right_â┐-Tubulin_unedited.tif]

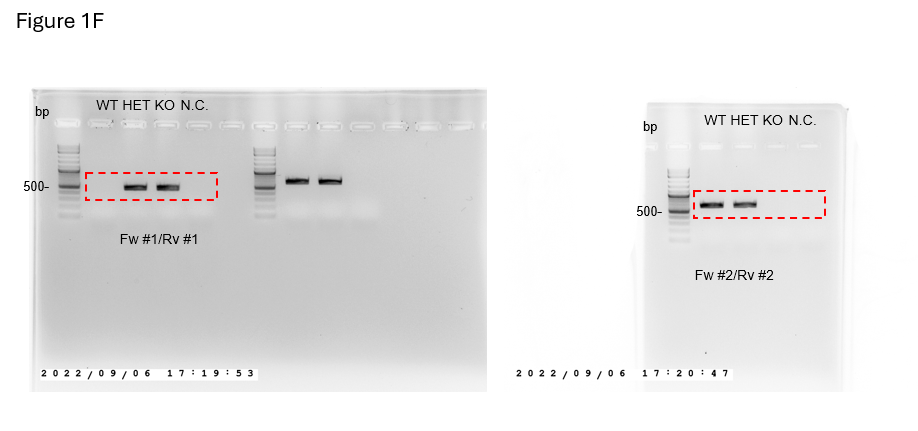

Supplement: Figure 1—source data 5. [file elife-92794-fig1-data5.zip › Figure 1-source data 5/Figure 1F_edited.tif]

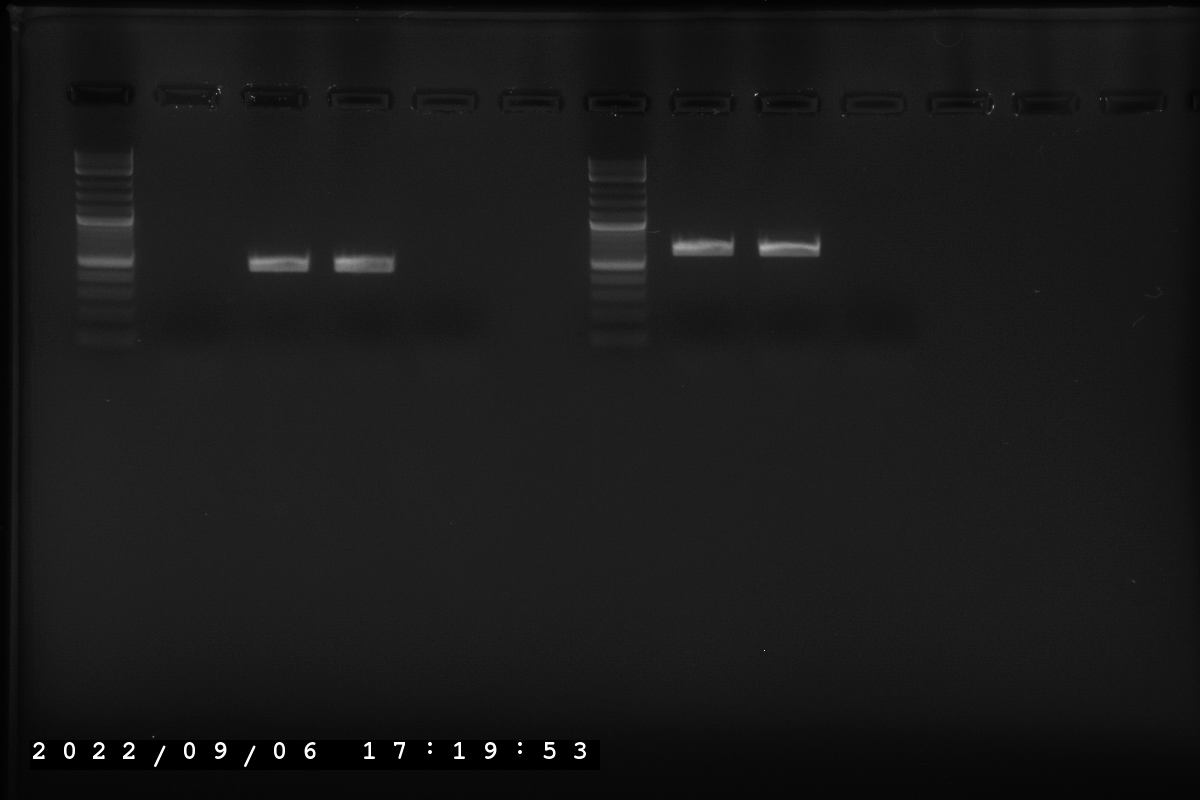

Supplement: Figure 1—source data 6. [file elife-92794-fig1-data6.zip › Figure 1-source data 6/Raw genotyping gel for Figure 1F_Fw 1 and Rv 1_unedited.tiff]

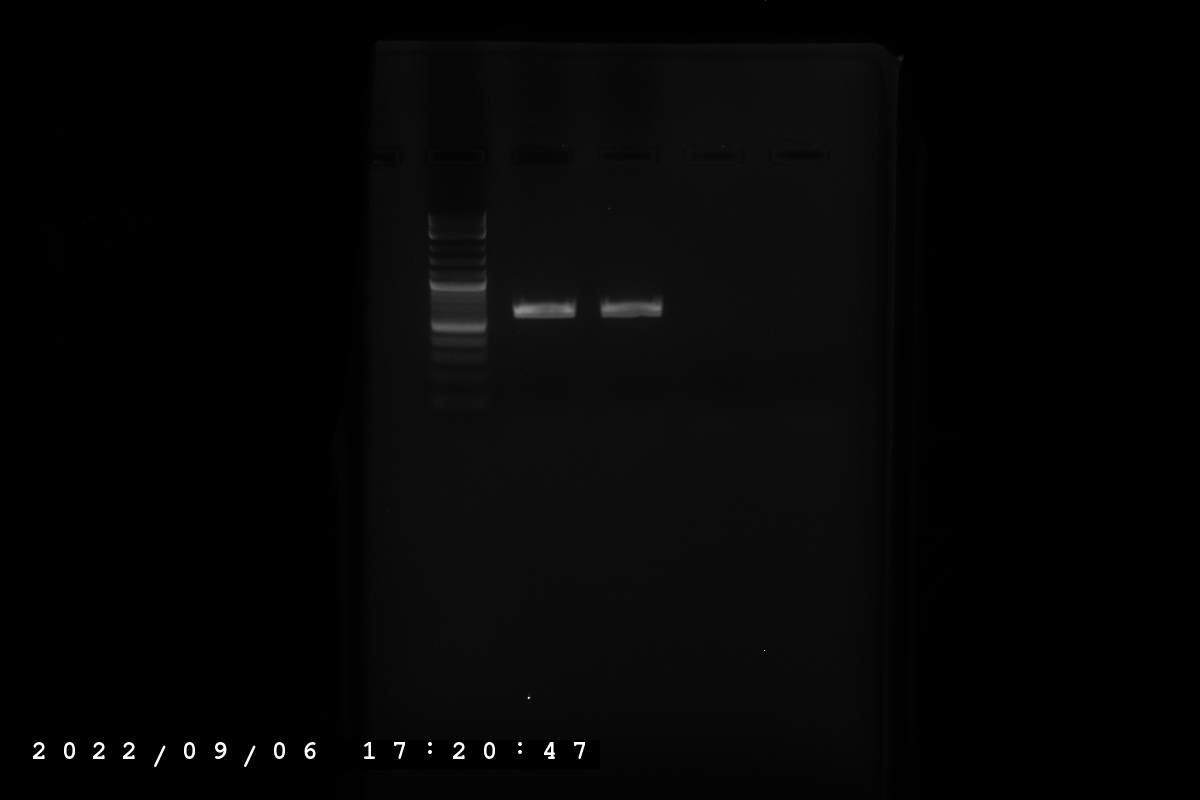

Supplement: Figure 1—source data 6. [file elife-92794-fig1-data6.zip › Figure 1-source data 6/Raw genotyping gel for Figure 1F_Fw 2 and Rv 2_unedited.tiff]

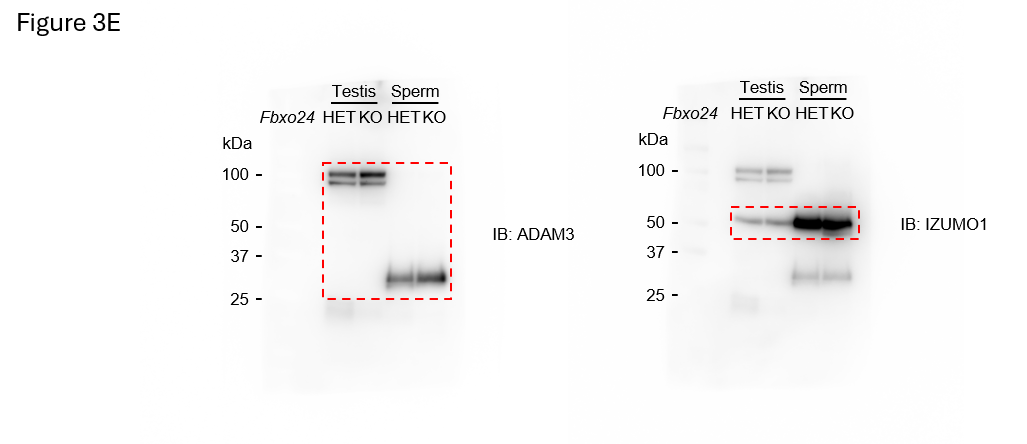

Supplement: Figure 3—source data 1. [file elife-92794-fig3-data1.zip › Figure 3-source data 1/Figure 3E_edited.tif]

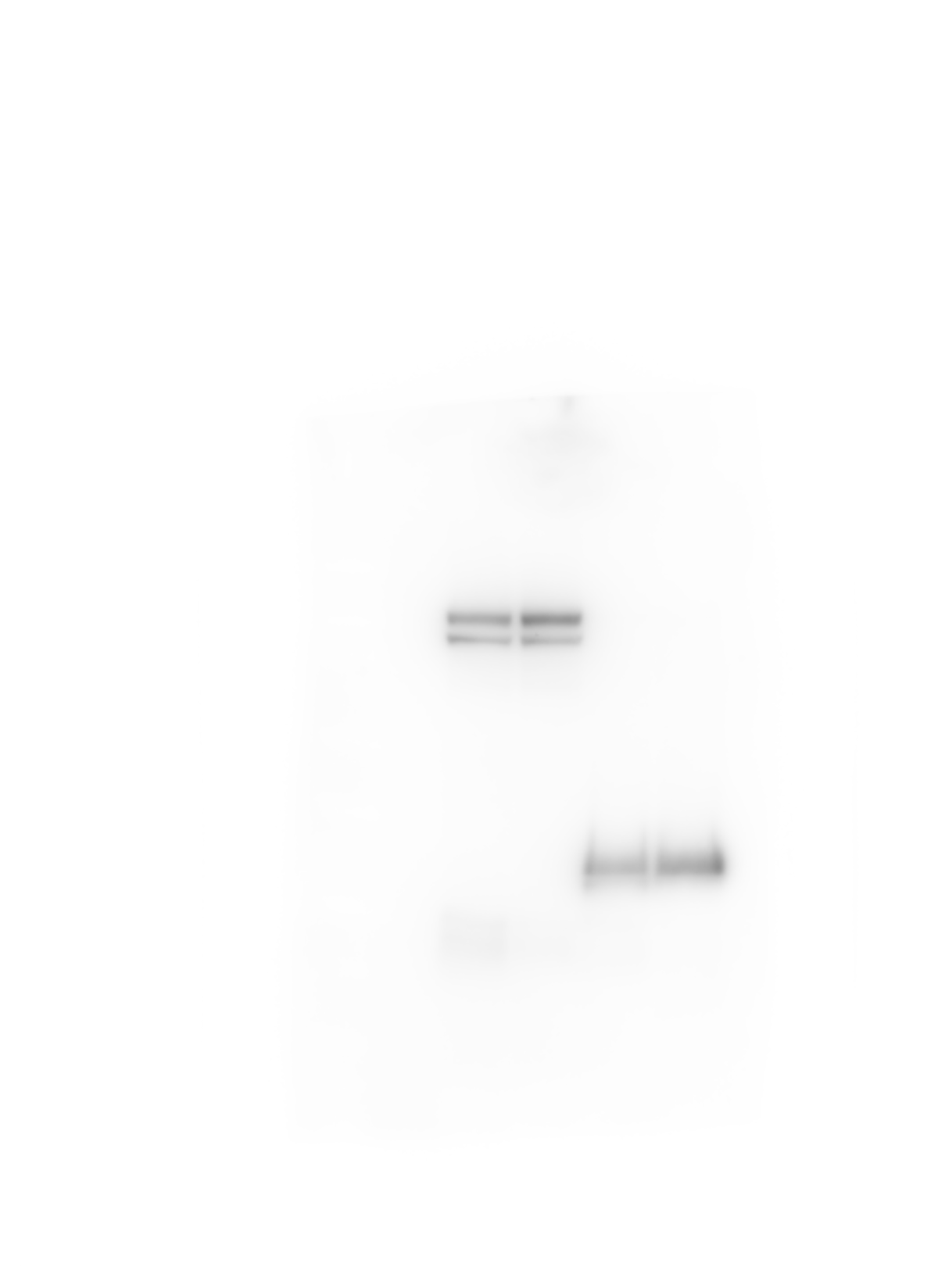

Supplement: Figure 3—source data 2. [file elife-92794-fig3-data2.zip › Figure 3-source data 2/Raw Western blot for Figure 3E_ADAM3_unedited.tif]

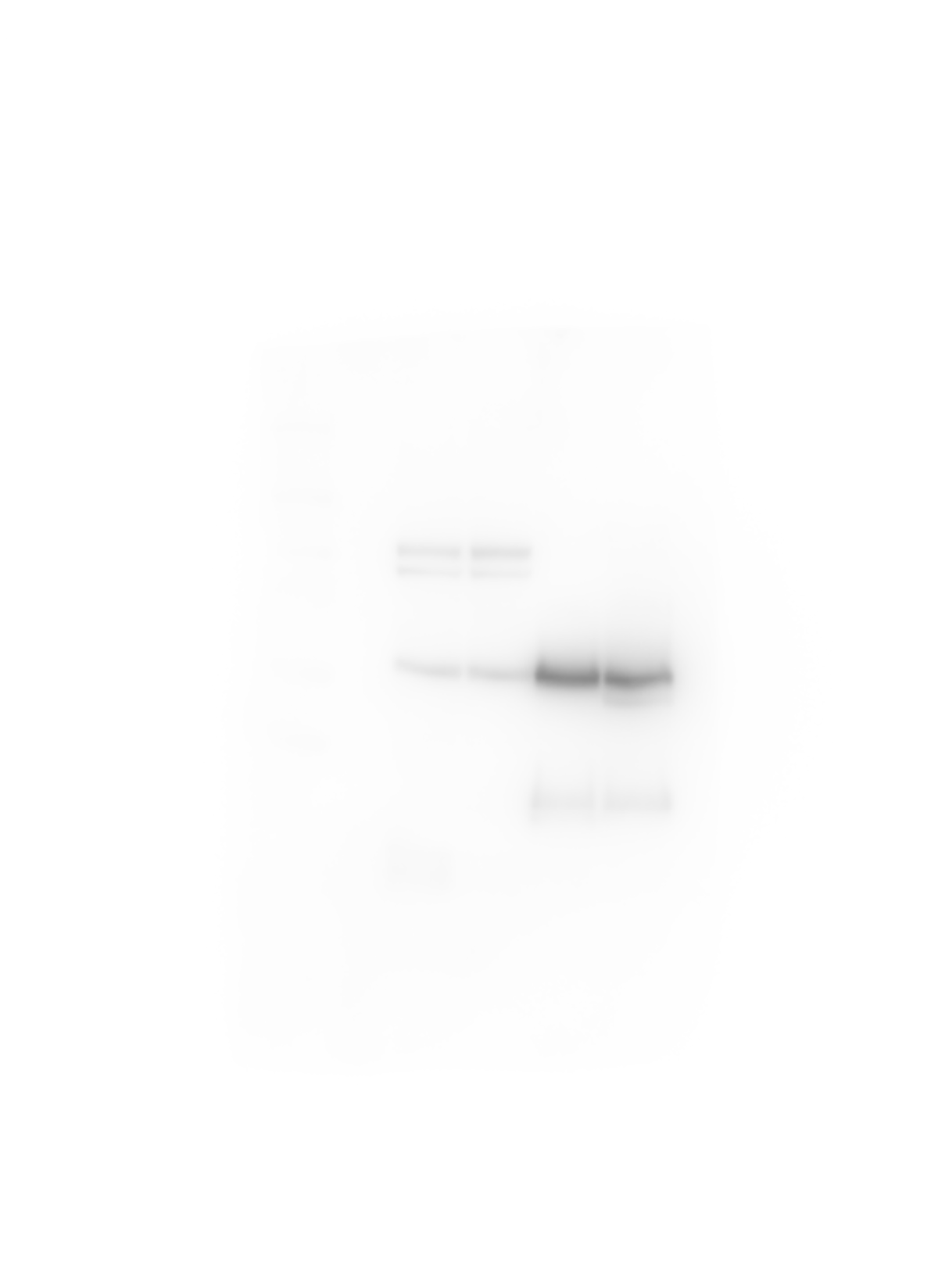

Supplement: Figure 3—source data 2. [file elife-92794-fig3-data2.zip › Figure 3-source data 2/Raw Western blot for Figure 3E_IZUMO1_unedited.tif]

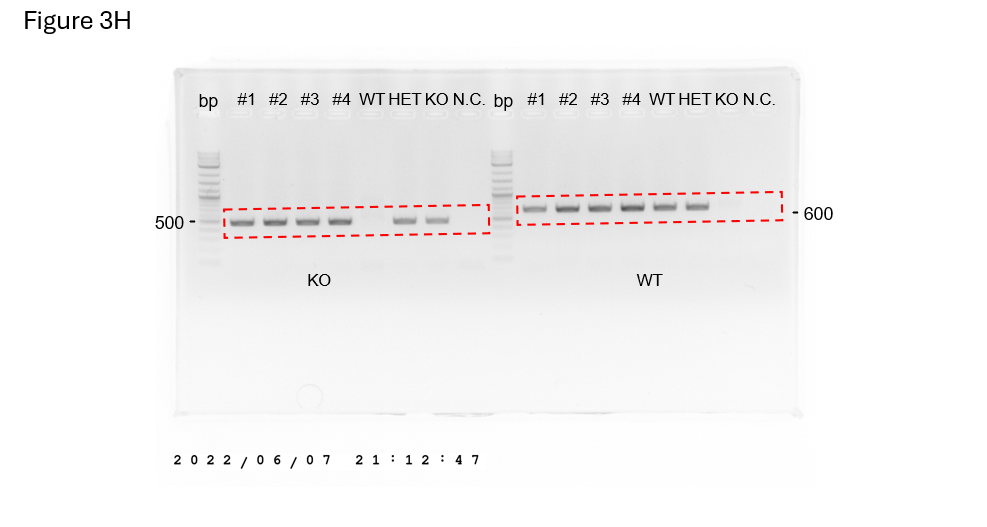

Supplement: Figure 3—source data 3. [file elife-92794-fig3-data3.zip › Figure 3-source data 3/Figure 3H_edited.tif]

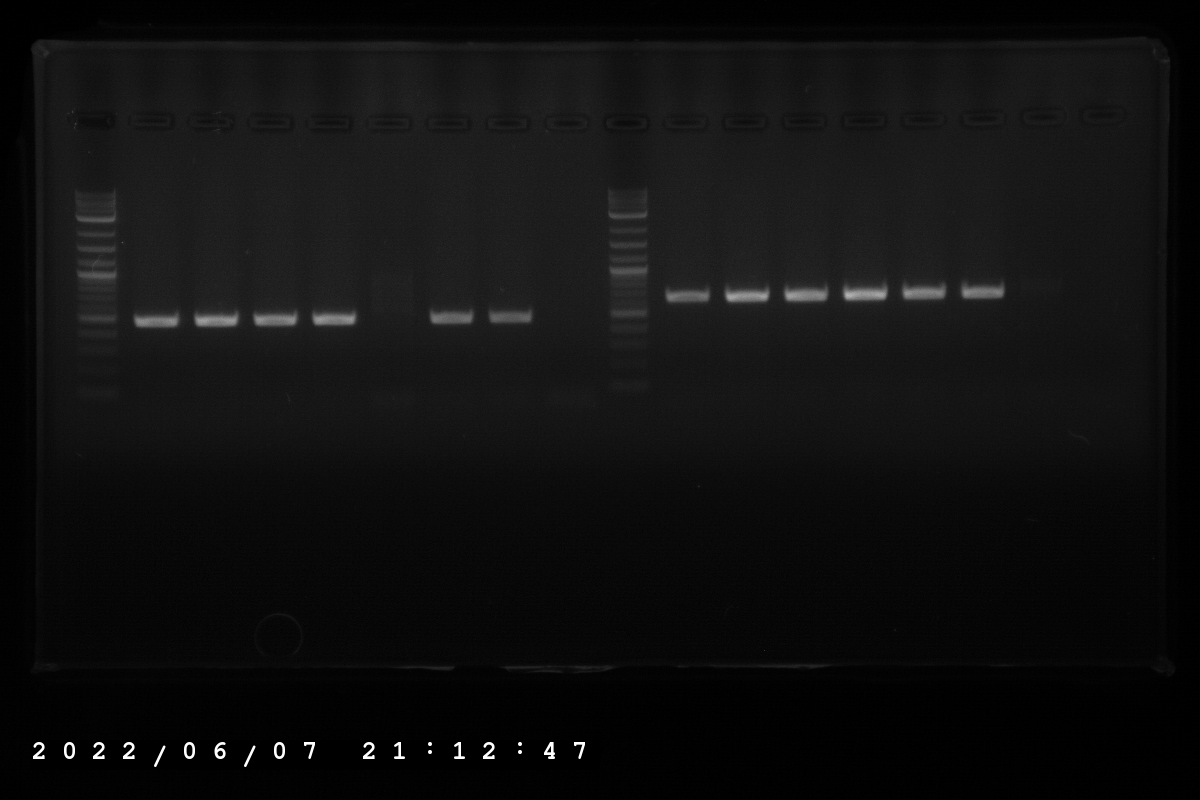

Supplement: Figure 3—source data 4. [file elife-92794-fig3-data4.zip › Figure 3-source data 4/Raw genotyping gel for Figure 3H_unedited.tiff]

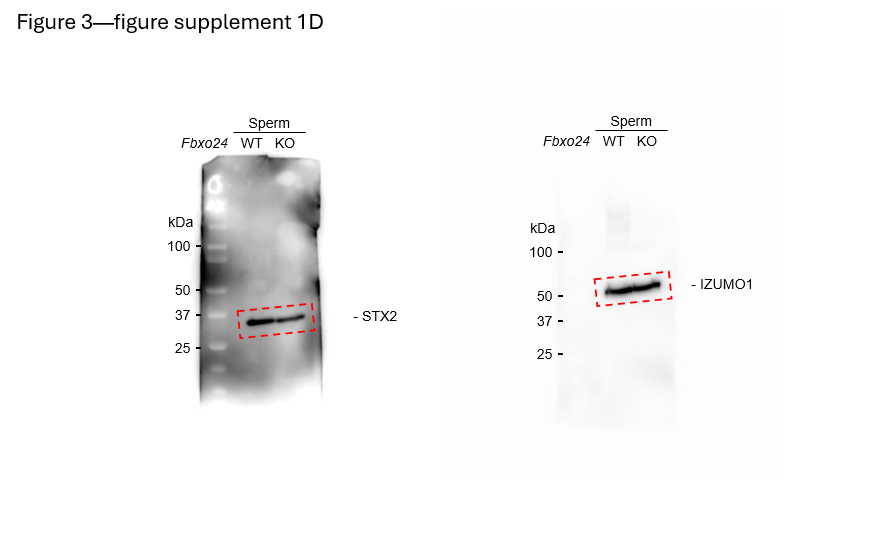

Supplement: Figure 3—figure supplement 1—source data 1. [file elife-92794-fig3-figsupp1-data1.zip › Figure 3-figure supplement 1-source data 1/Figure 3-figure supplement 1D_edited.tif]

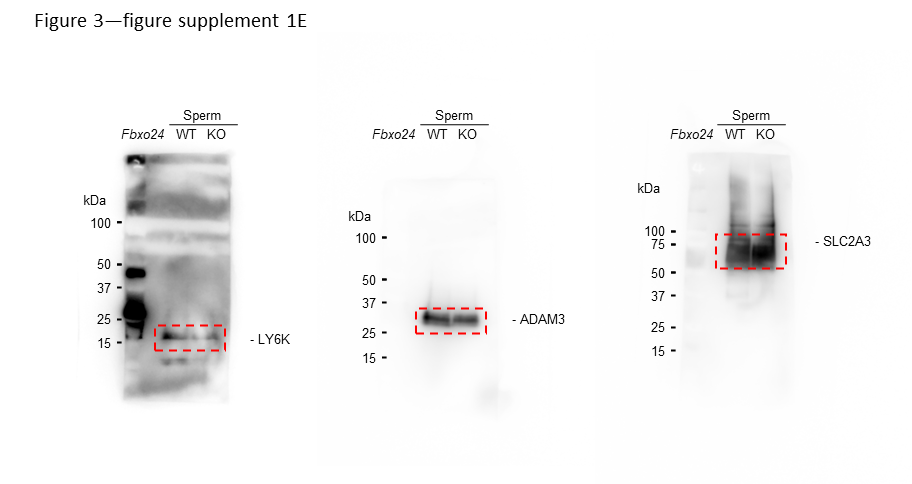

Supplement: Figure 3—figure supplement 1—source data 1. [file elife-92794-fig3-figsupp1-data1.zip › Figure 3-figure supplement 1-source data 1/Figure 3-figure supplement 1E_edited.tif]

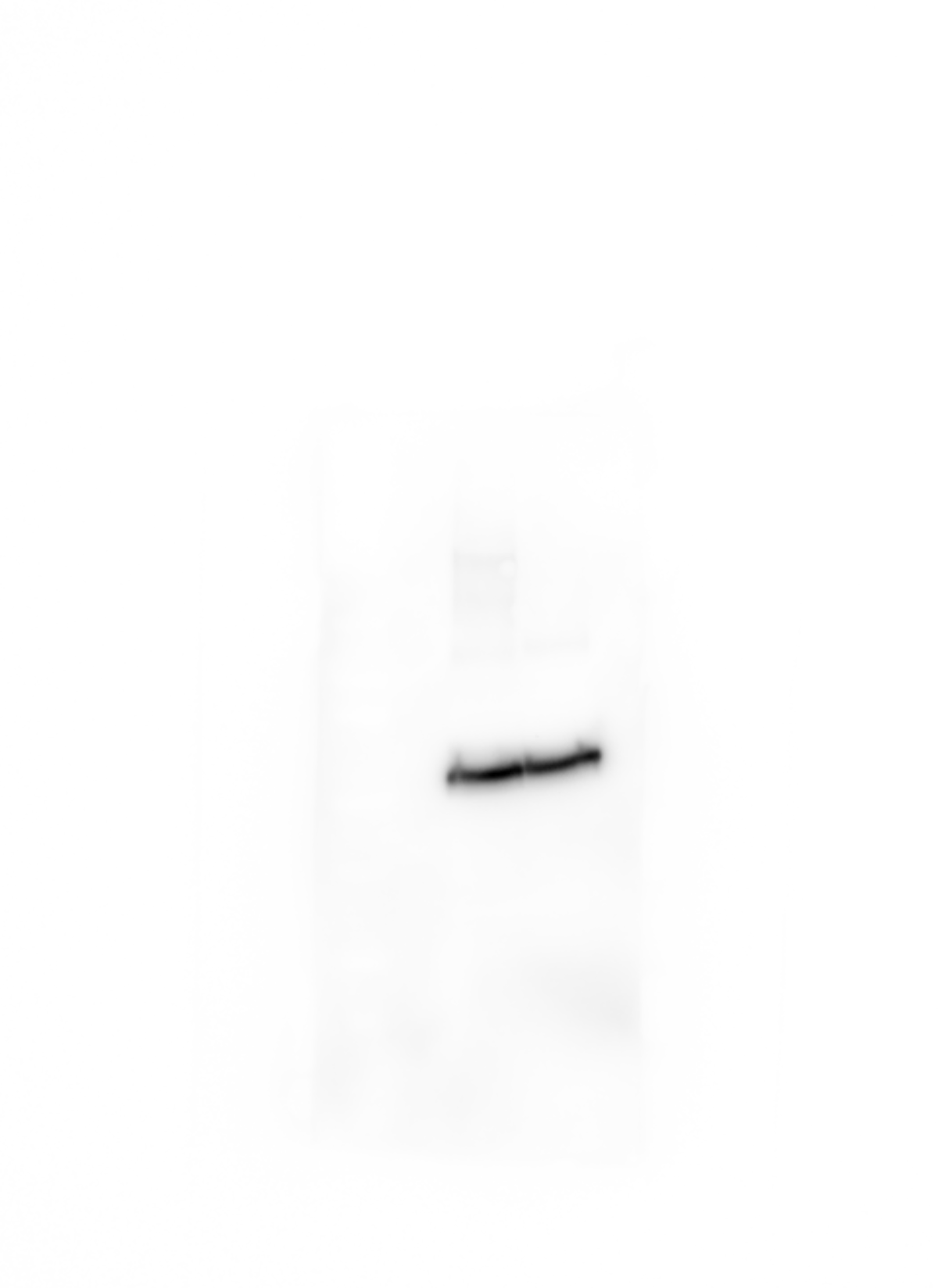

Supplement: Figure 3—figure supplement 1—source data 2. [file elife-92794-fig3-figsupp1-data2.zip › Figure 3-figure supplement 1-source data 2/Raw western blot for Figure 3-figure supplement 1D_IZUMO1_unedited.tif]

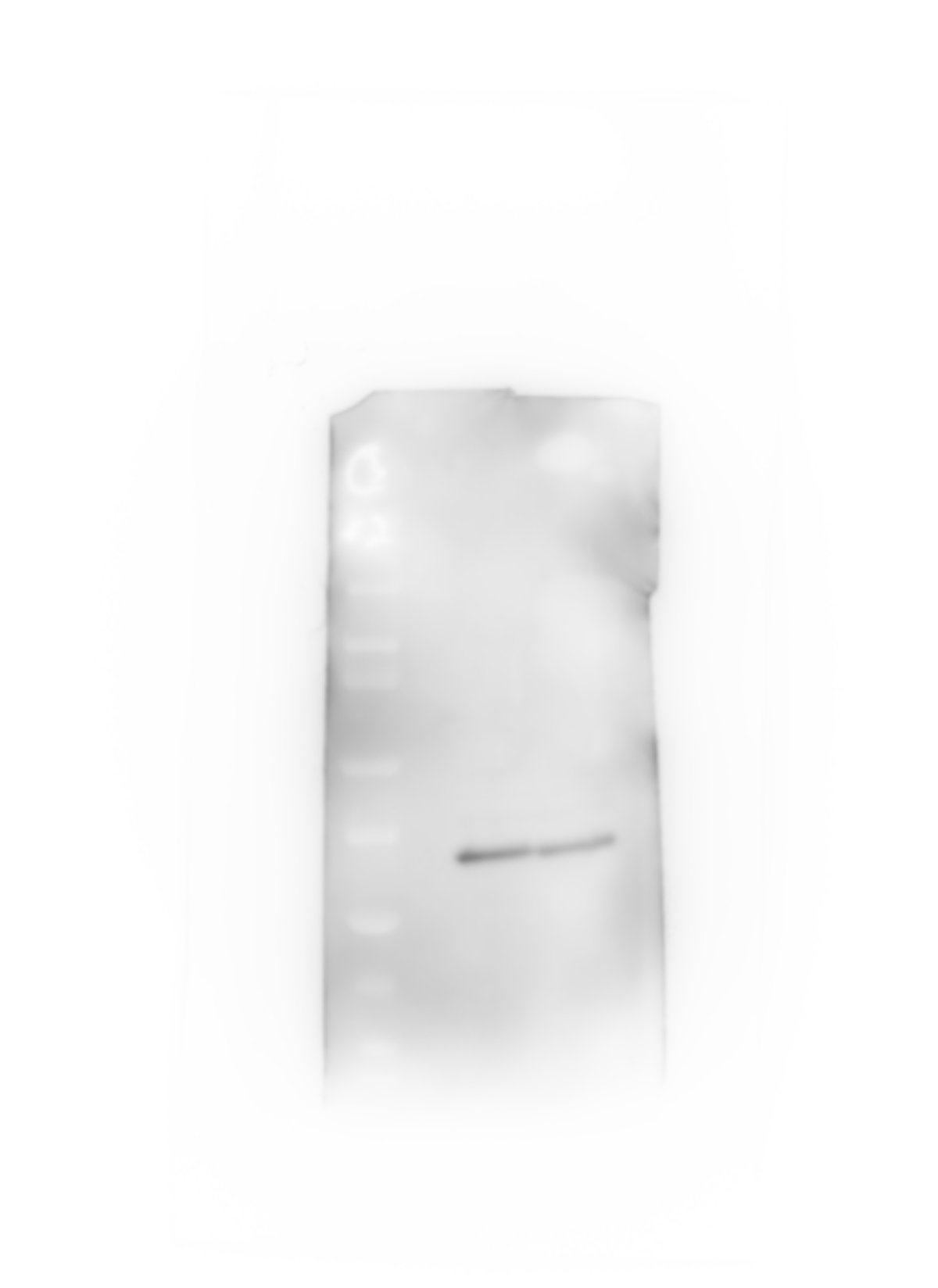

Supplement: Figure 3—figure supplement 1—source data 2. [file elife-92794-fig3-figsupp1-data2.zip › Figure 3-figure supplement 1-source data 2/Raw western blot for Figure 3-figure supplement 1D_STX2_unedited.tif]

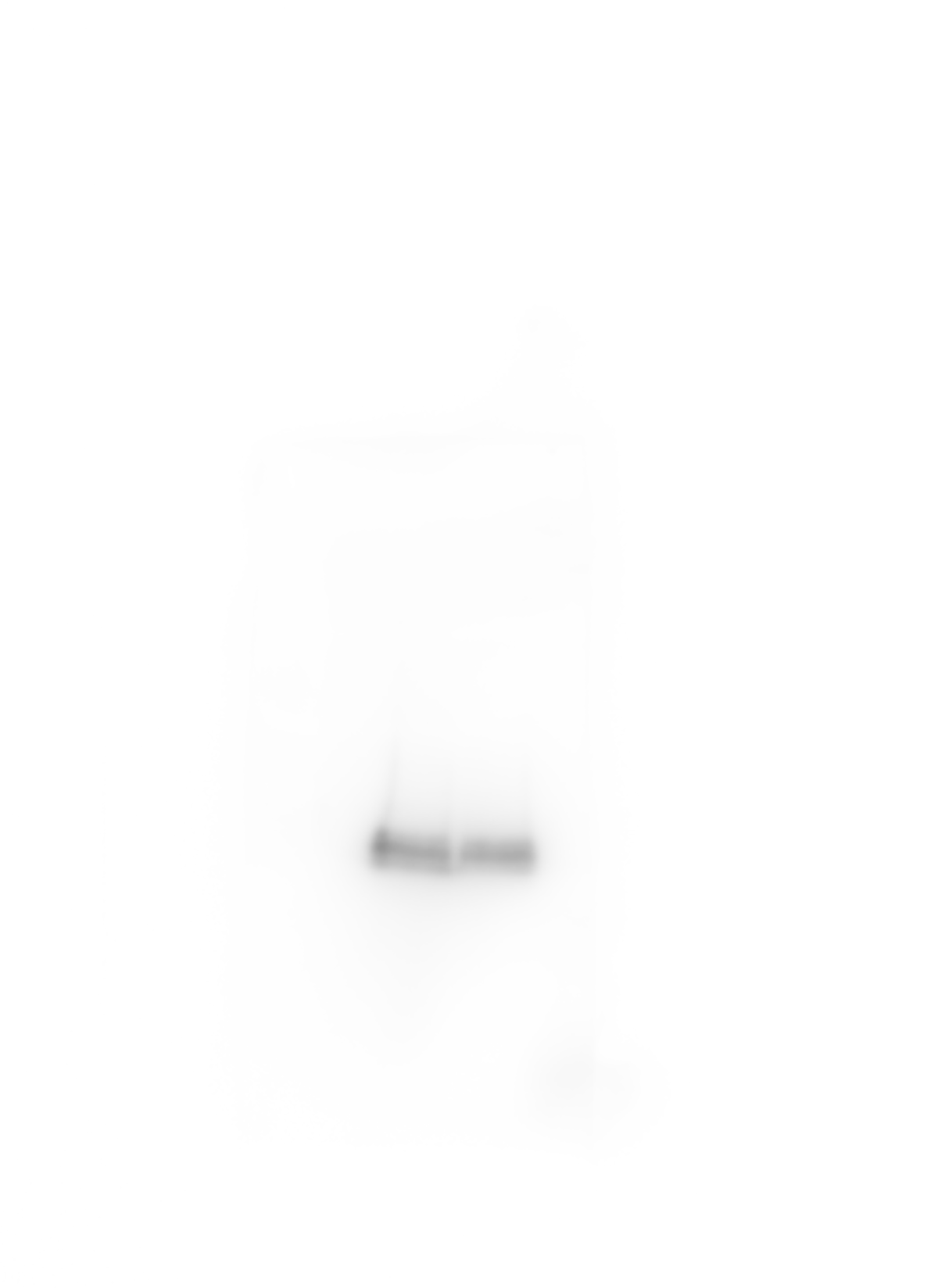

Supplement: Figure 3—figure supplement 1—source data 2. [file elife-92794-fig3-figsupp1-data2.zip › Figure 3-figure supplement 1-source data 2/Raw western blot for Figure 3-figure supplement 1E_ADAM3_unedited.tif]

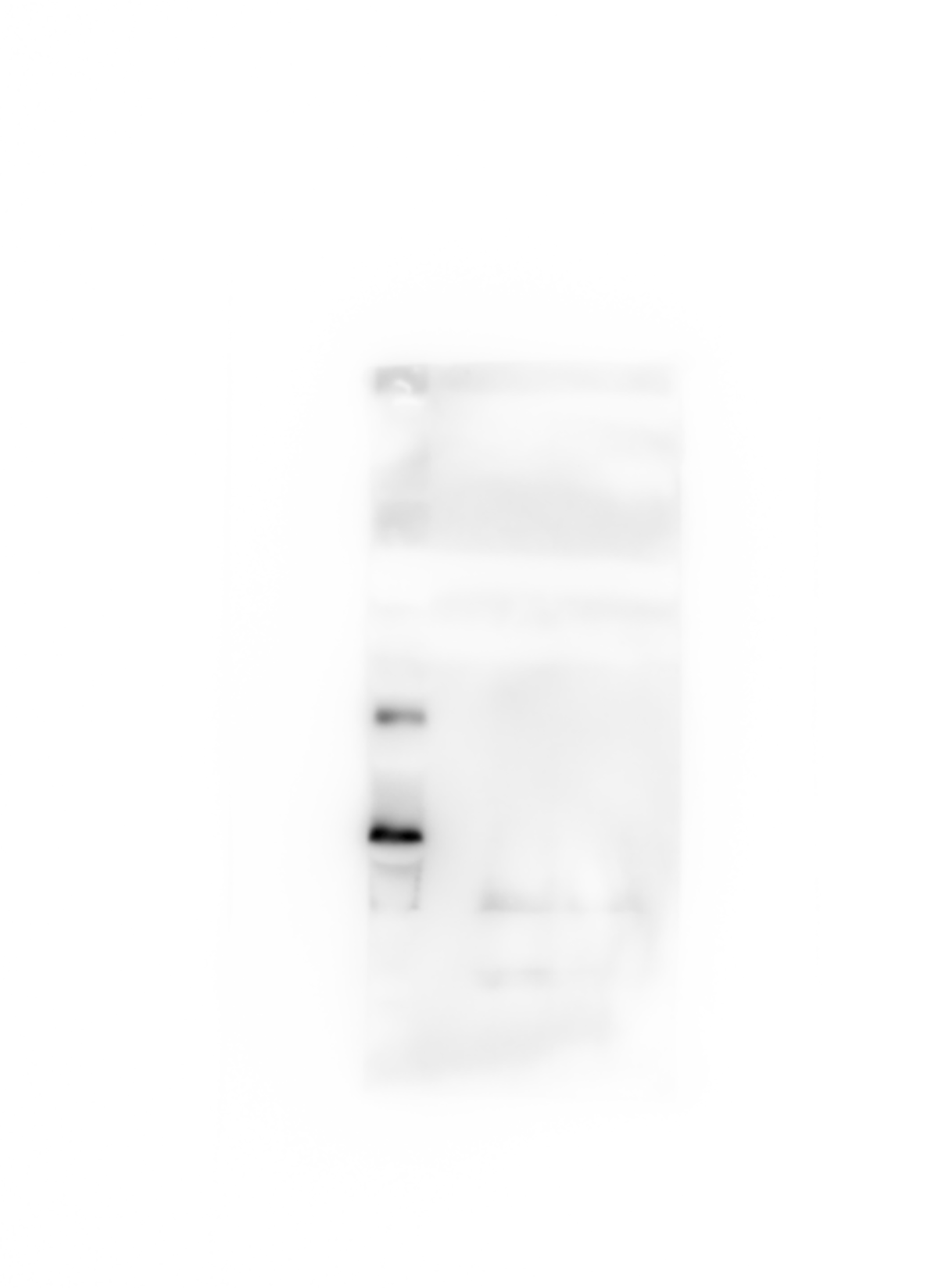

Supplement: Figure 3—figure supplement 1—source data 2. [file elife-92794-fig3-figsupp1-data2.zip › Figure 3-figure supplement 1-source data 2/Raw western blot for Figure 3-figure supplement 1E_LY6K_unedited.tif]

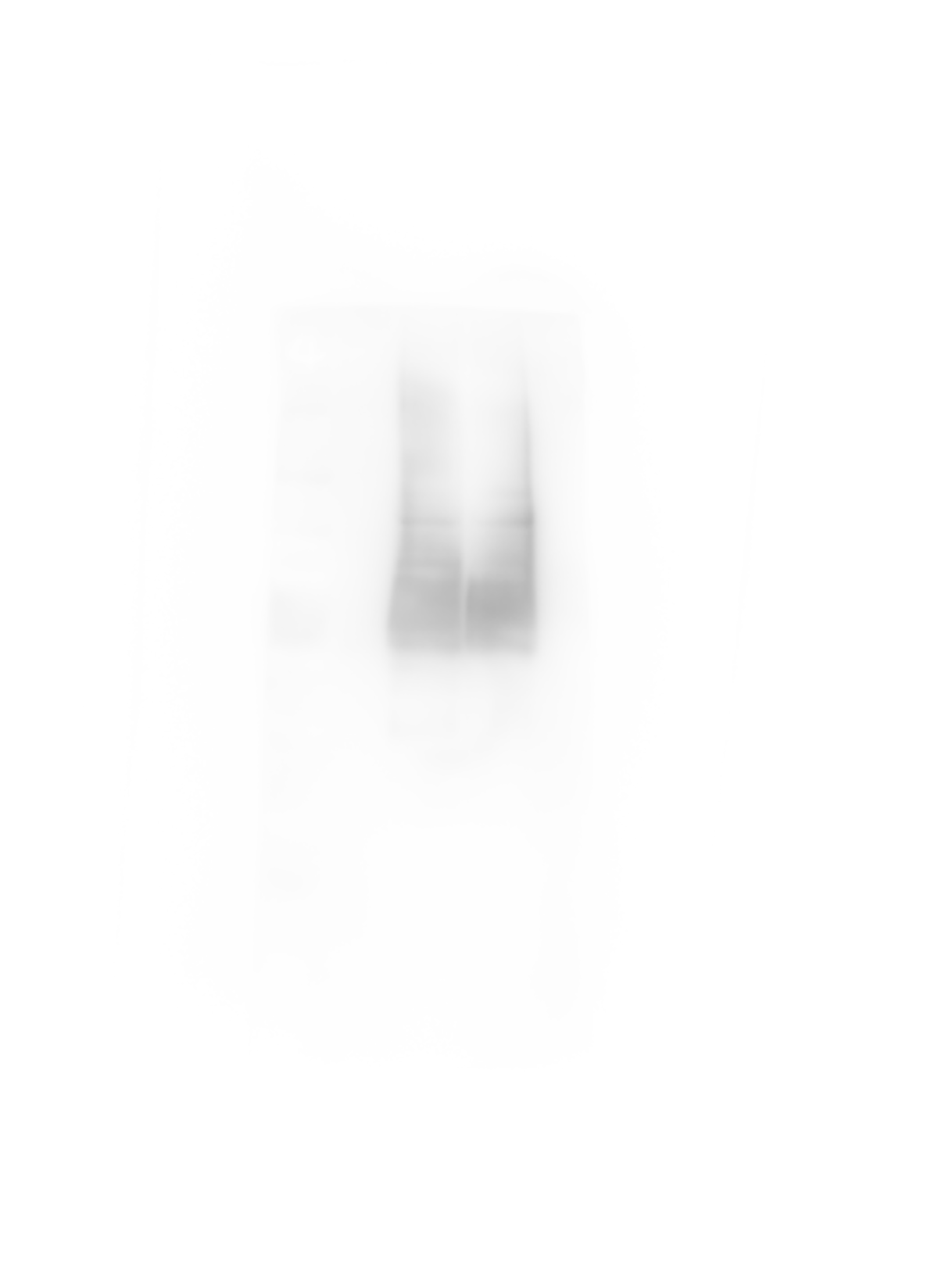

Supplement: Figure 3—figure supplement 1—source data 2. [file elife-92794-fig3-figsupp1-data2.zip › Figure 3-figure supplement 1-source data 2/Raw western blot for Figure 3-figure supplement 1E_SLC2A3_unedited.tif]

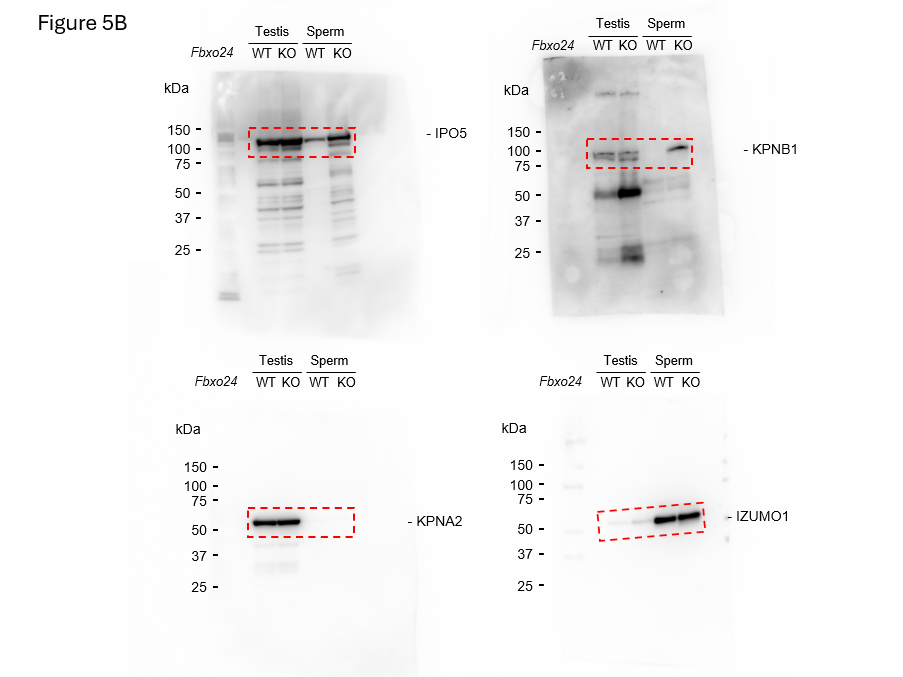

Supplement: Figure 5—source data 1. [file elife-92794-fig5-data1.zip › Figure 5-source data 1/Figure 5B_edited.tif]

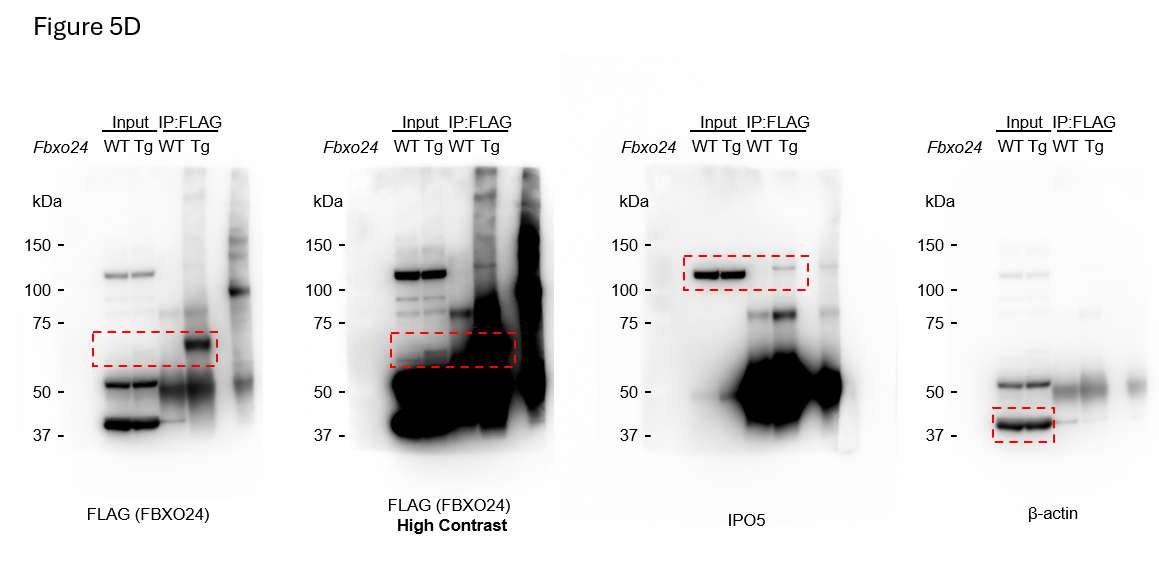

Supplement: Figure 5—source data 1. [file elife-92794-fig5-data1.zip › Figure 5-source data 1/Figure 5D_edited.tif]

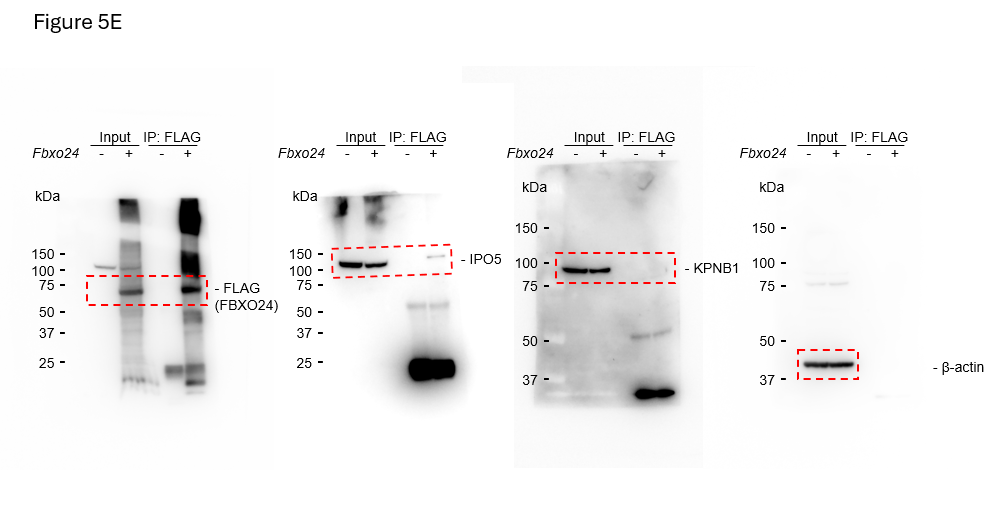

Supplement: Figure 5—source data 1. [file elife-92794-fig5-data1.zip › Figure 5-source data 1/Figure 5E_edited.tif]

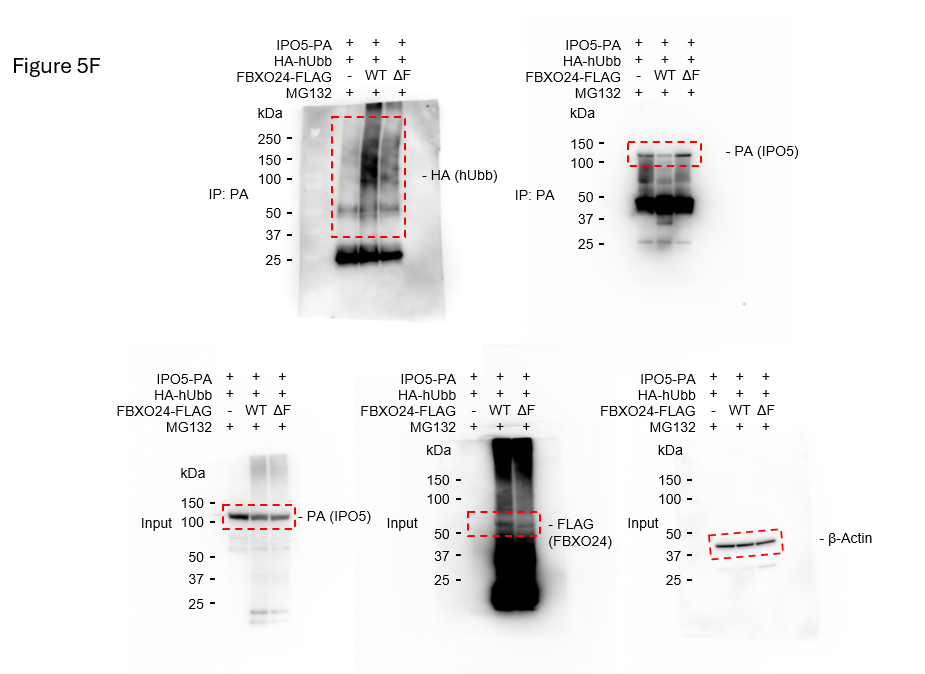

Supplement: Figure 5—source data 1. [file elife-92794-fig5-data1.zip › Figure 5-source data 1/Figure 5F_edited.tif]

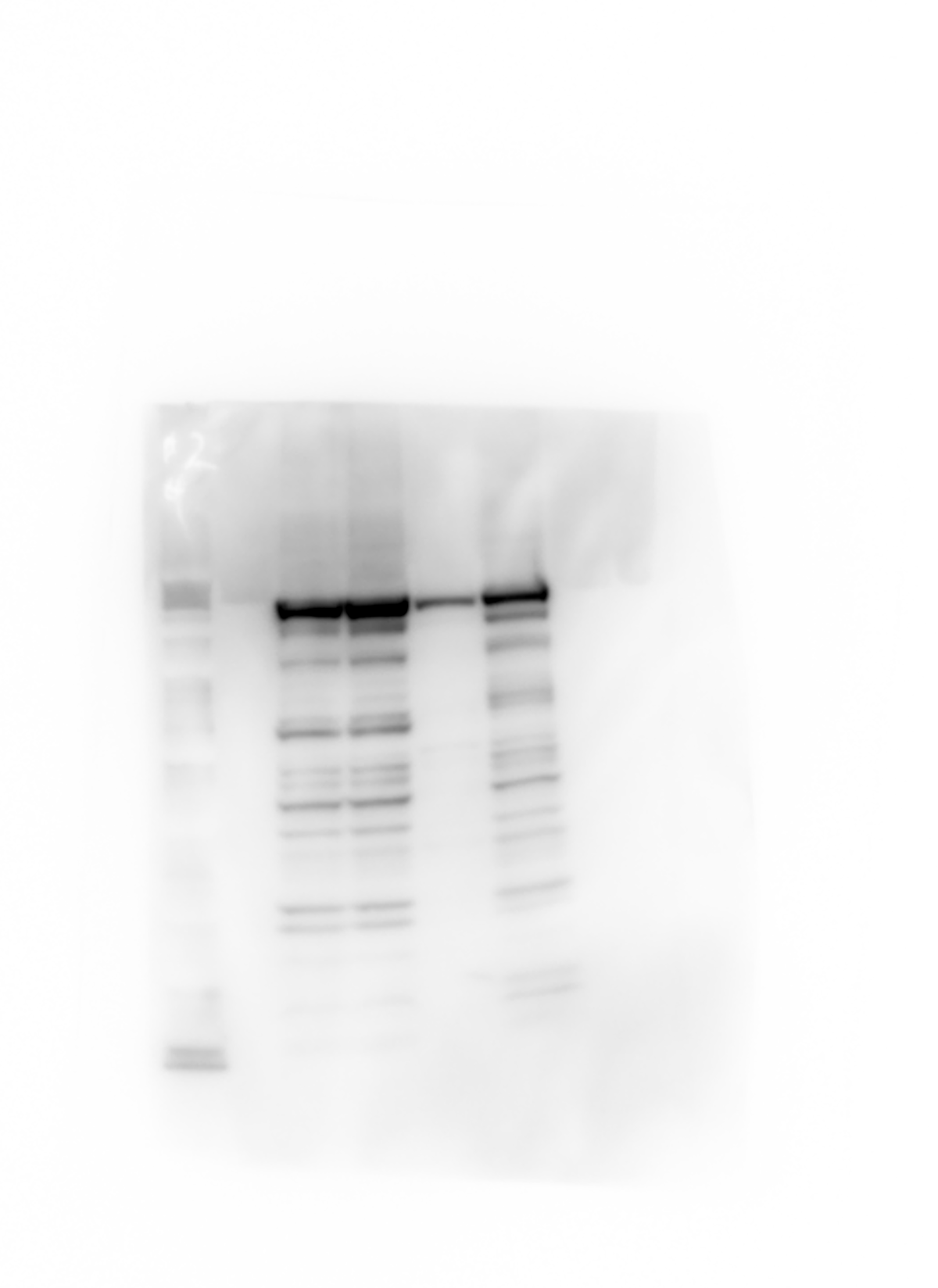

Supplement: Figure 5—source data 2. [file elife-92794-fig5-data2.zip › Figure 5-source data 2/Raw Western blot for Figure 5B_IPO5_unedited.tif]

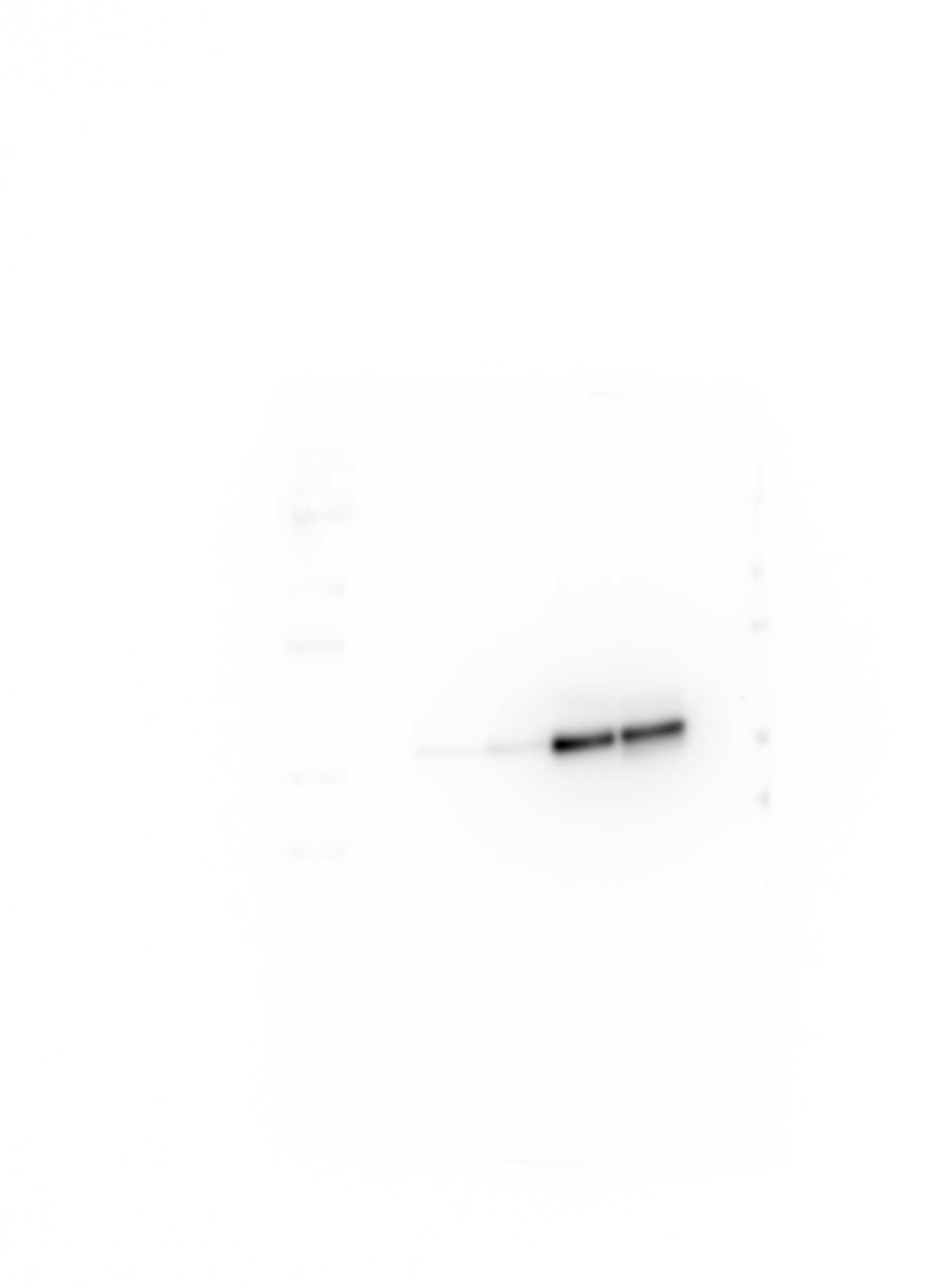

Supplement: Figure 5—source data 2. [file elife-92794-fig5-data2.zip › Figure 5-source data 2/Raw Western blot for Figure 5B_IZUMO1_unedited.tif]

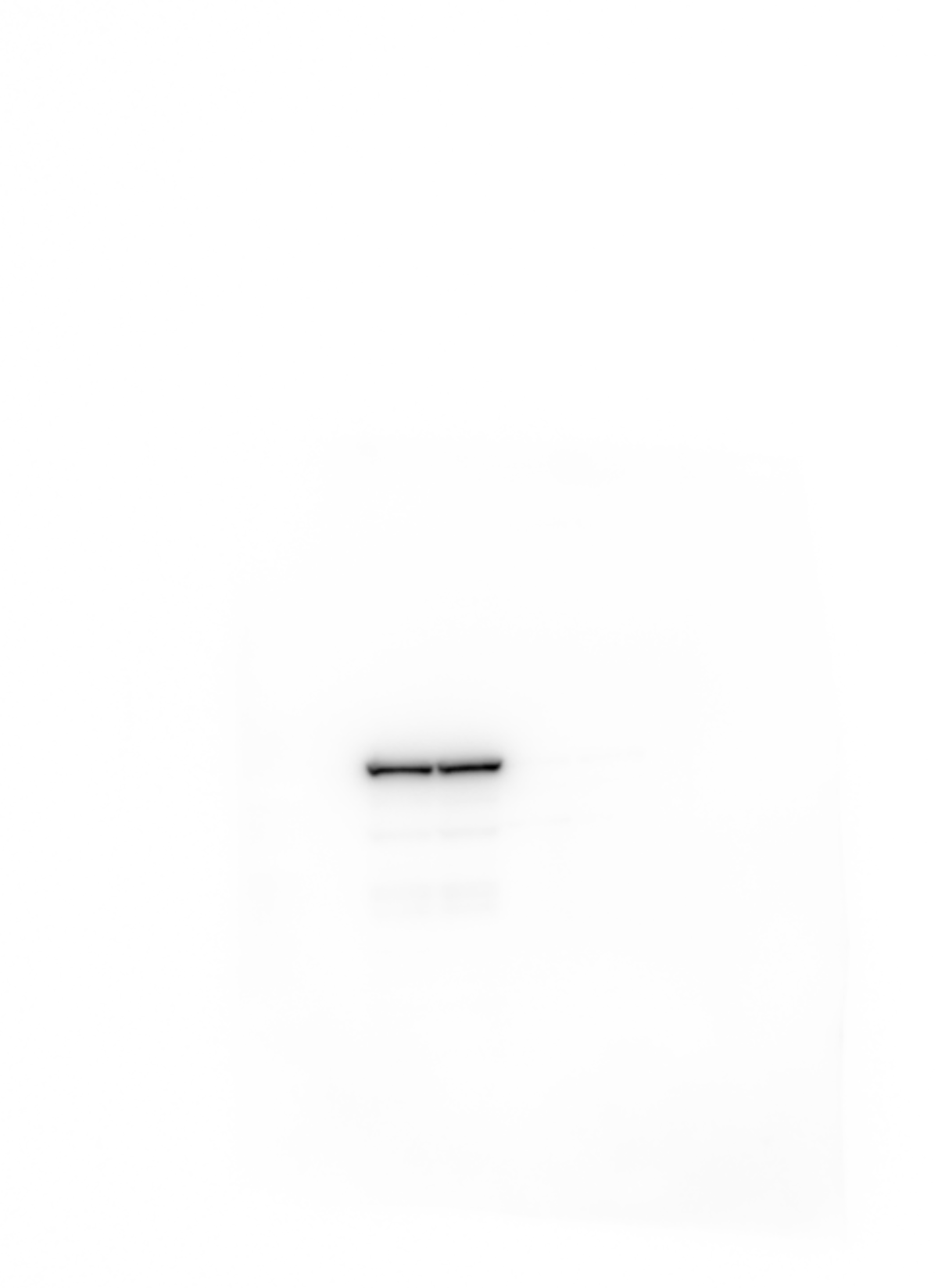

Supplement: Figure 5—source data 2. [file elife-92794-fig5-data2.zip › Figure 5-source data 2/Raw Western blot for Figure 5B_KPNA2_unedited.tif]

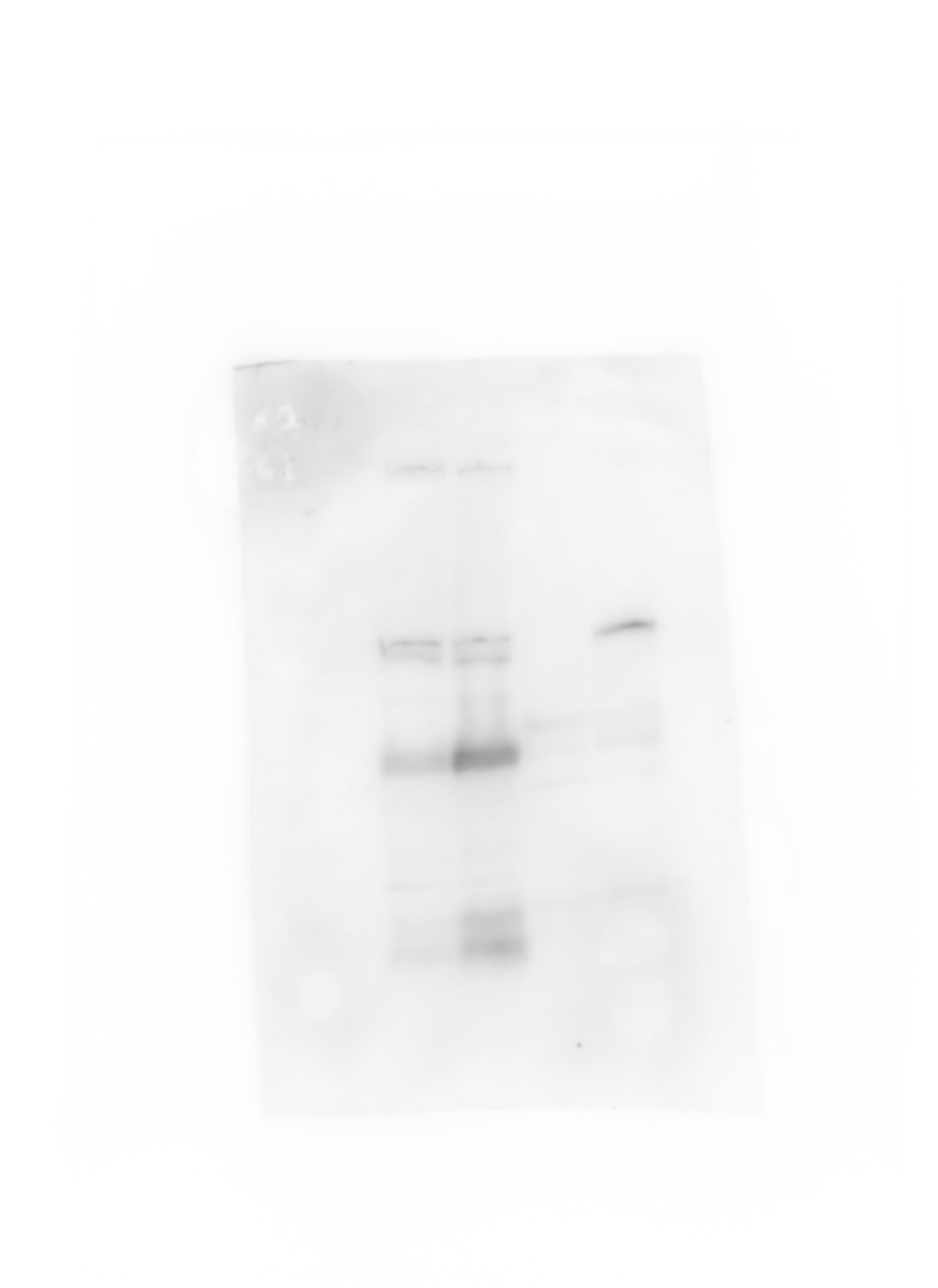

Supplement: Figure 5—source data 2. [file elife-92794-fig5-data2.zip › Figure 5-source data 2/Raw Western blot for Figure 5B_KPNB1_unedited.tif]

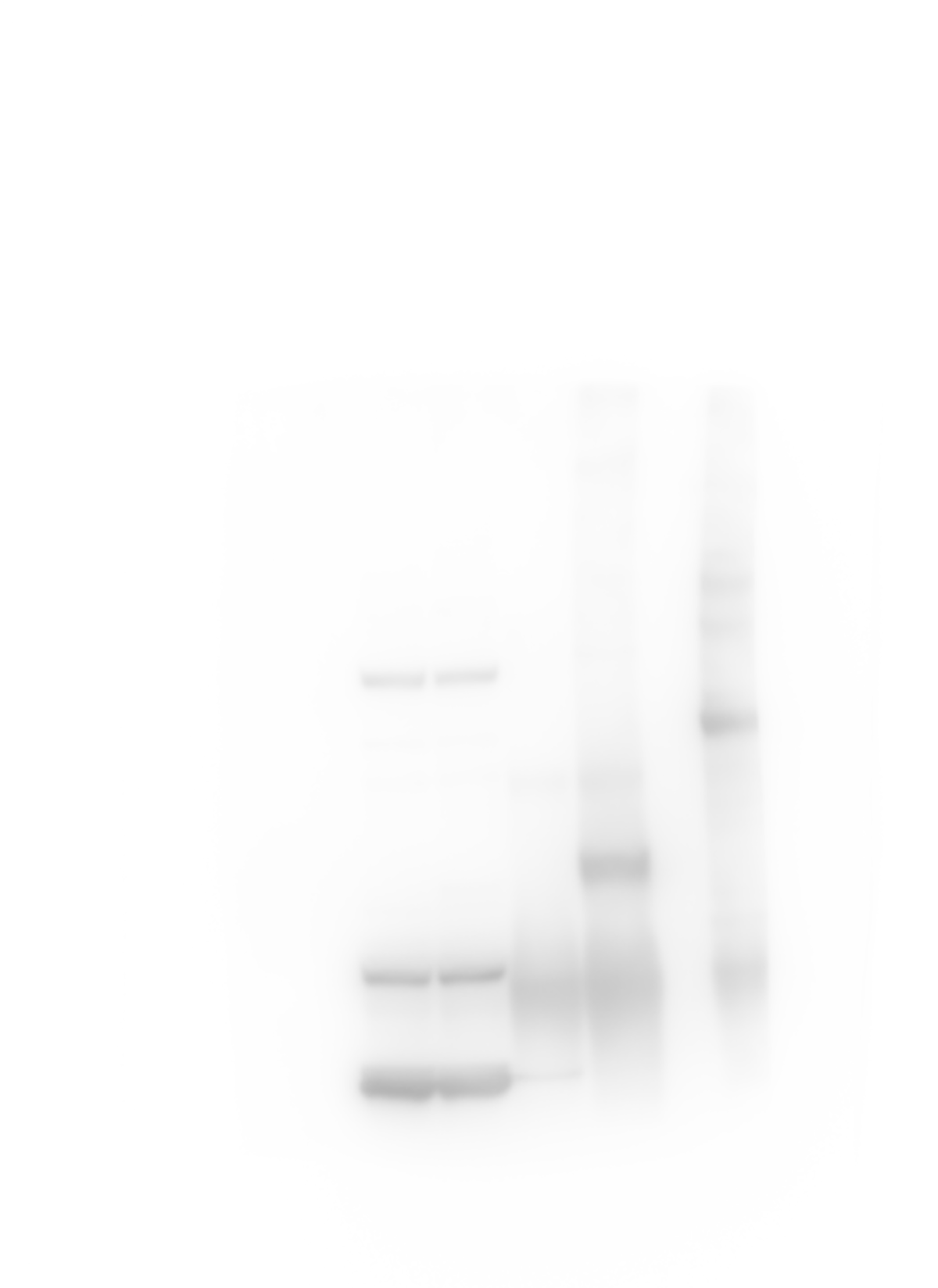

Supplement: Figure 5—source data 2. [file elife-92794-fig5-data2.zip › Figure 5-source data 2/Raw Western blot for Figure 5D_FLAG_unedited.tif]

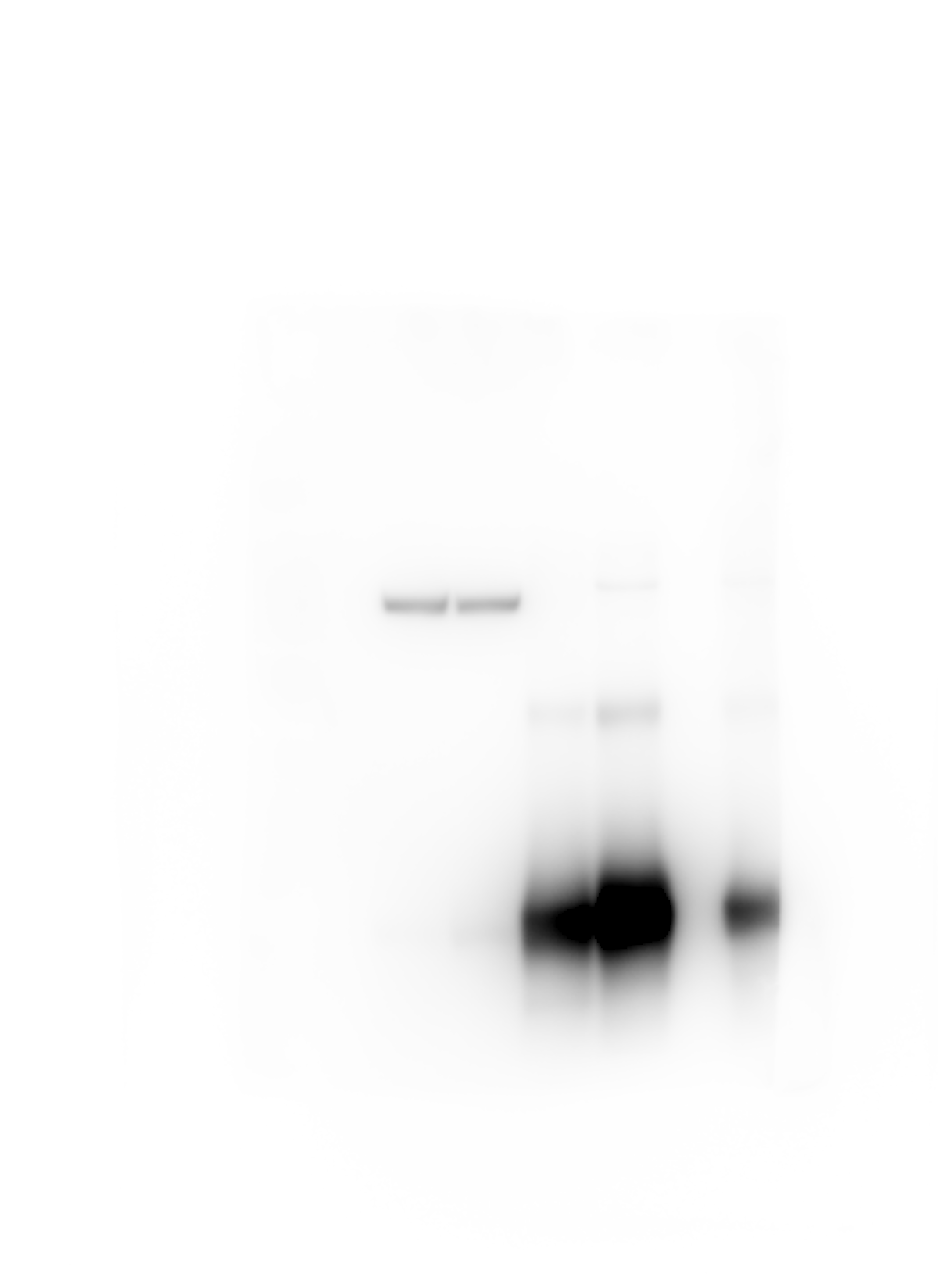

Supplement: Figure 5—source data 2. [file elife-92794-fig5-data2.zip › Figure 5-source data 2/Raw Western blot for Figure 5D_IPO5_unedited.tif]

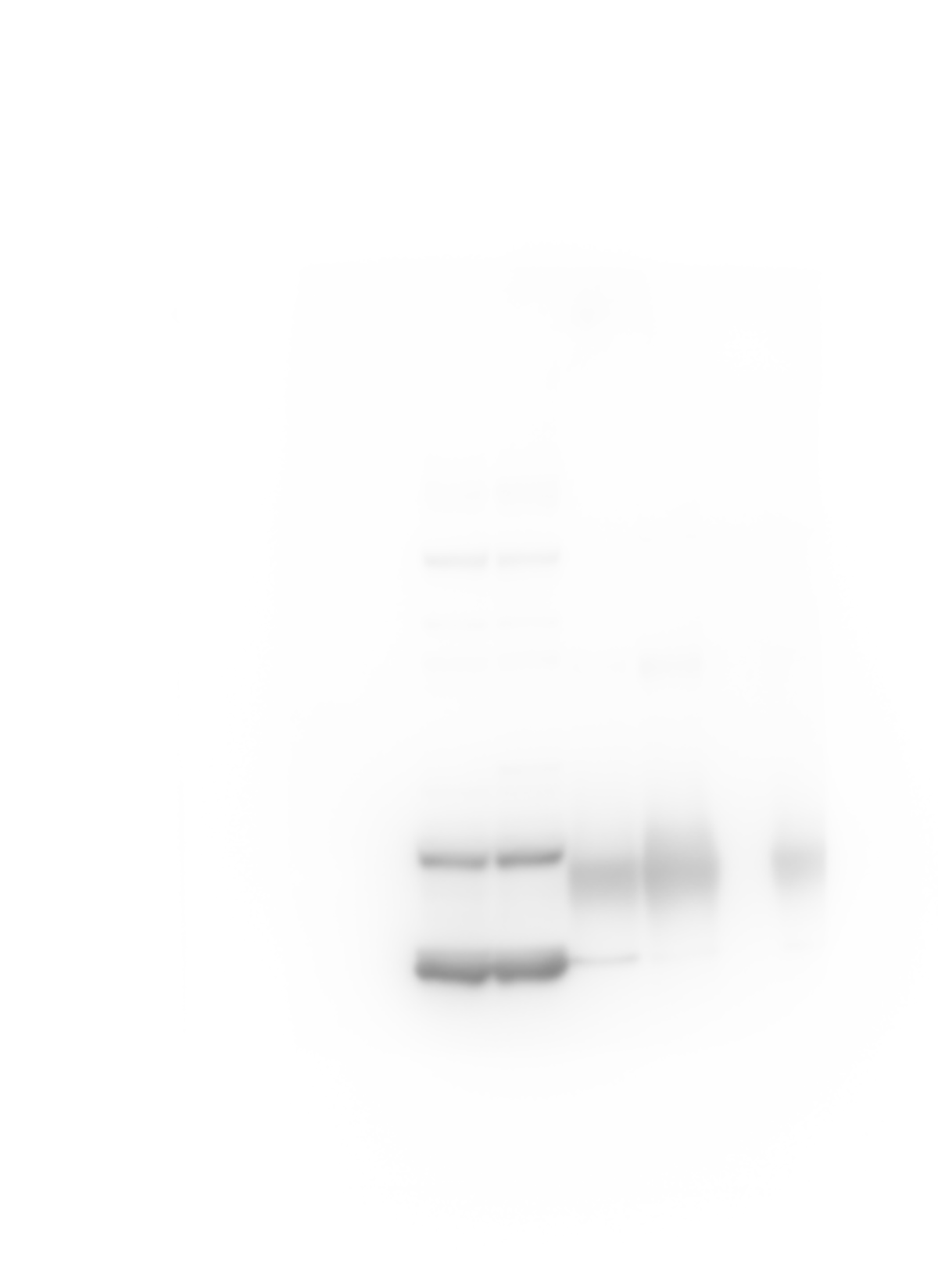

Supplement: Figure 5—source data 2. [file elife-92794-fig5-data2.zip › Figure 5-source data 2/Raw Western blot for Figure 5D_â└-actin_unedited.tif]

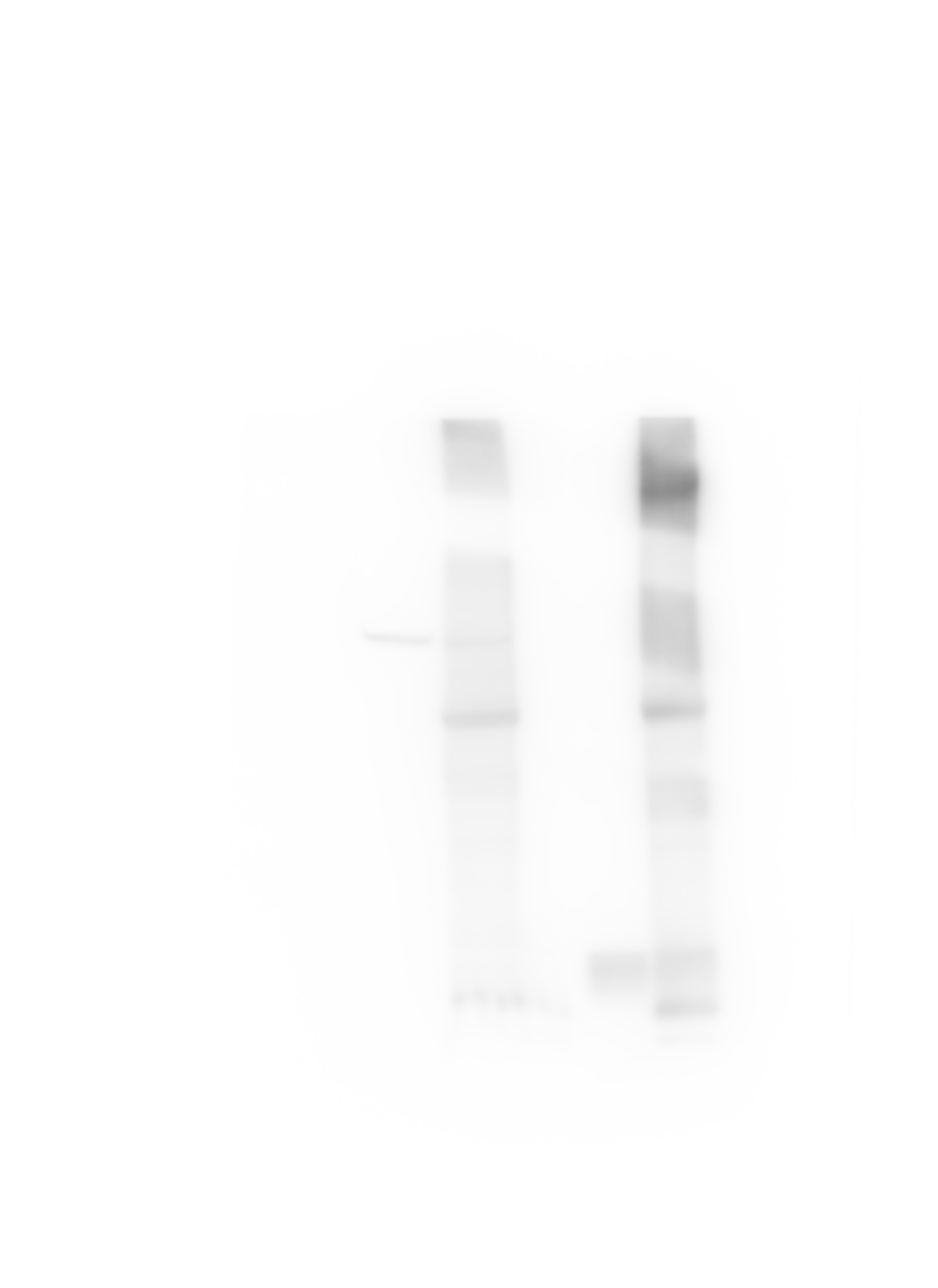

Supplement: Figure 5—source data 2. [file elife-92794-fig5-data2.zip › Figure 5-source data 2/Raw Western blot for Figure 5E_FLAG_unedited.tif]

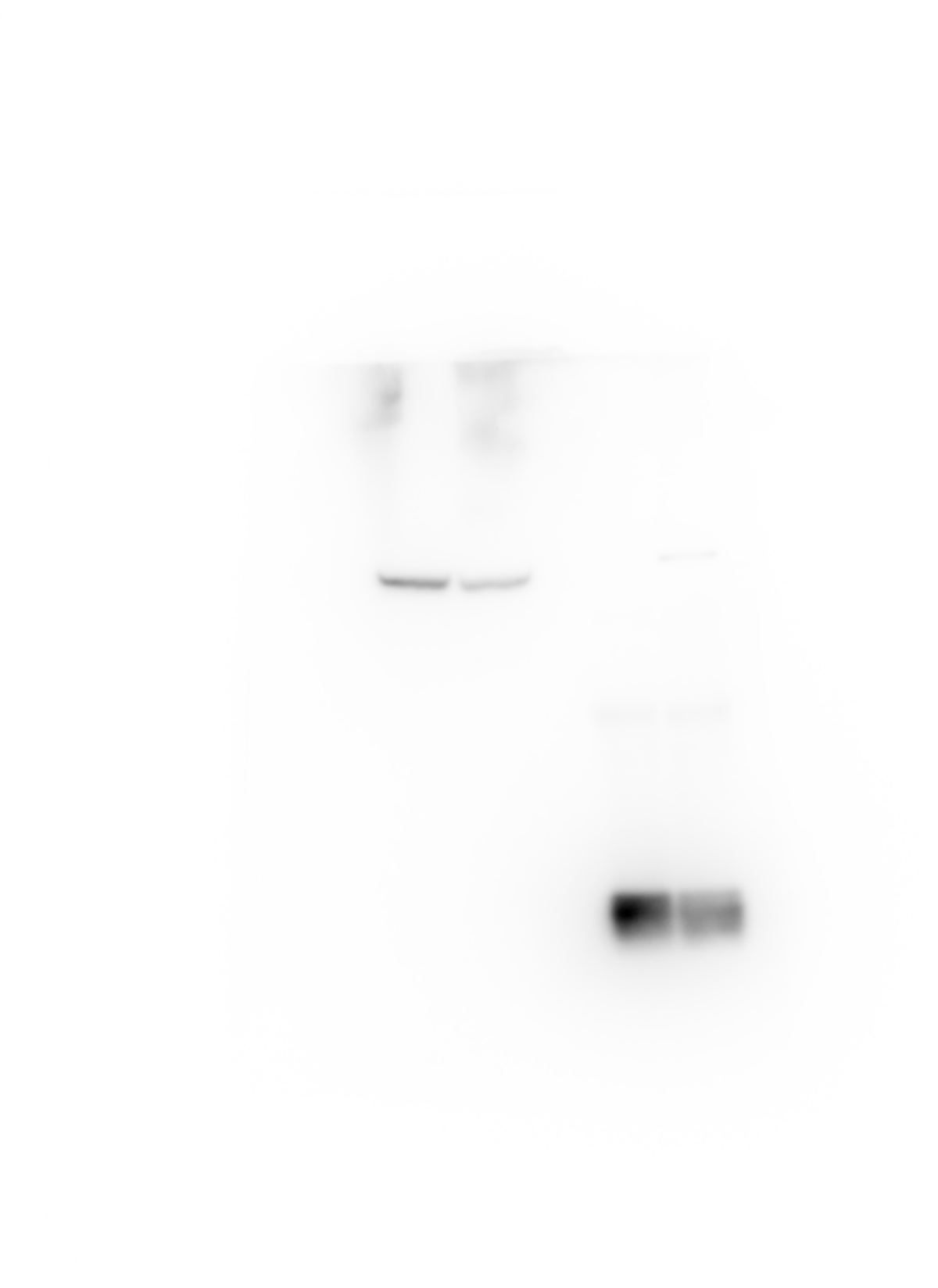

Supplement: Figure 5—source data 2. [file elife-92794-fig5-data2.zip › Figure 5-source data 2/Raw Western blot for Figure 5E_IPO5_unedited.tif]

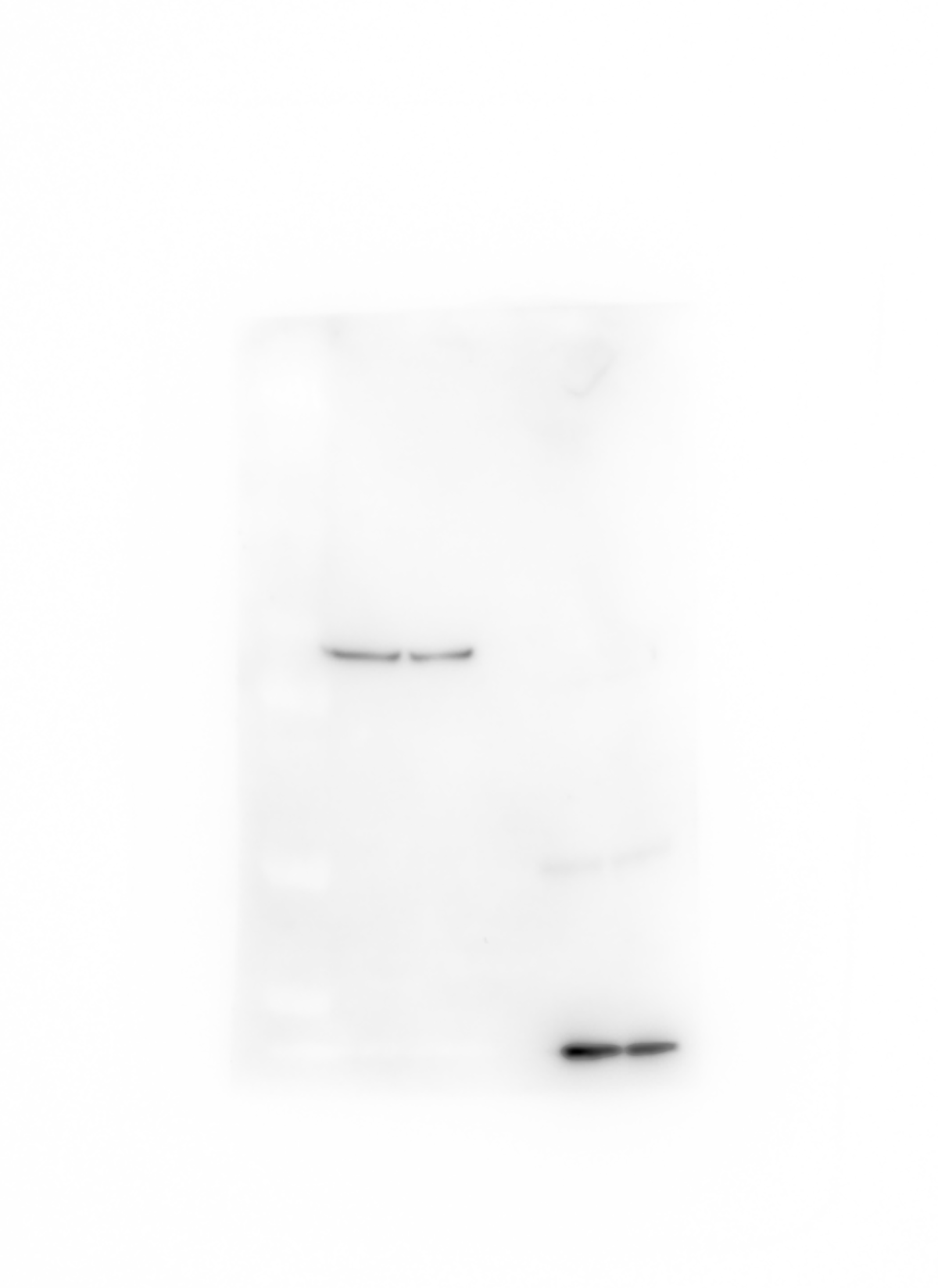

Supplement: Figure 5—source data 2. [file elife-92794-fig5-data2.zip › Figure 5-source data 2/Raw Western blot for Figure 5E_KPNB1_unedited.tif]

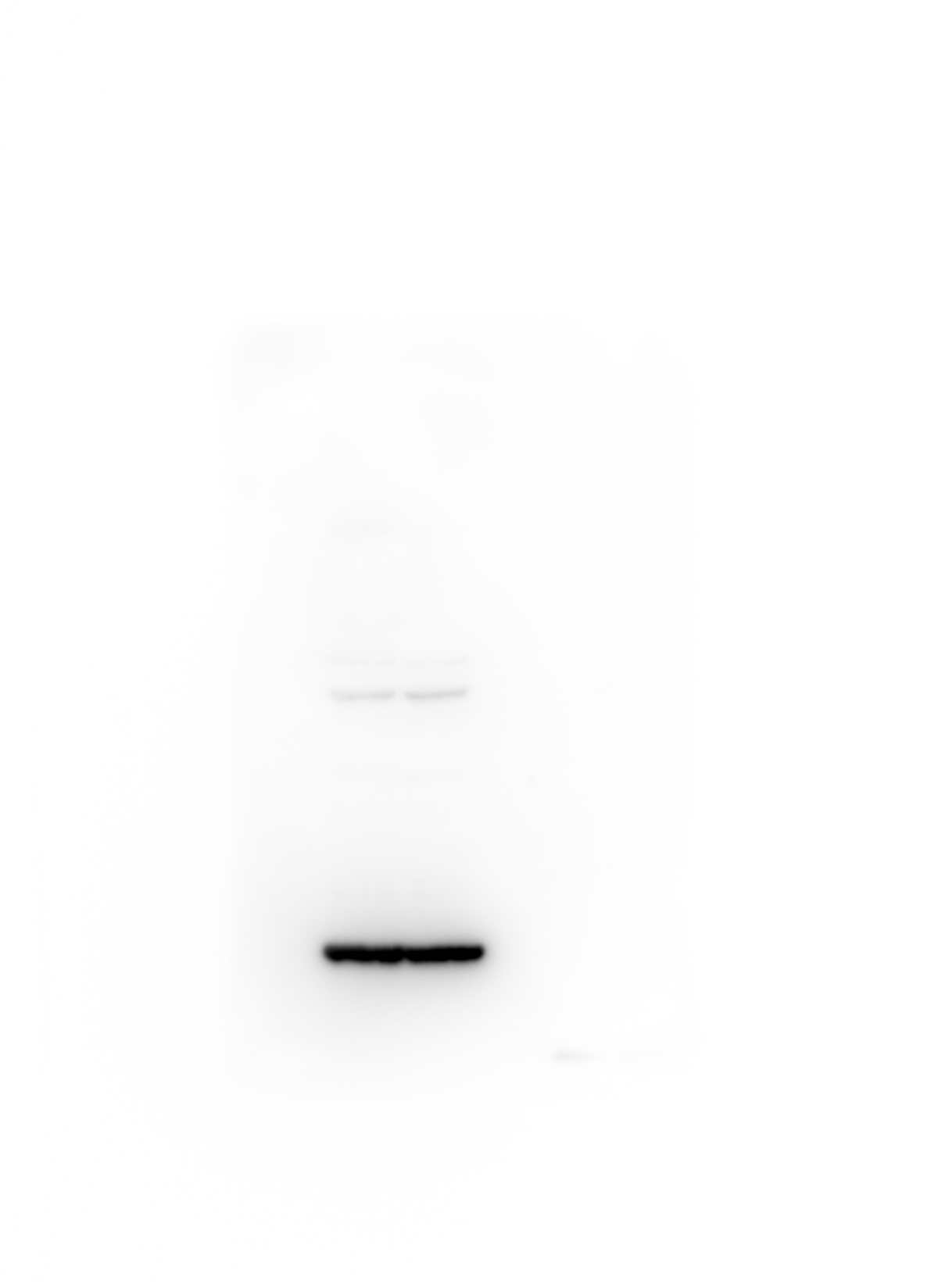

Supplement: Figure 5—source data 2. [file elife-92794-fig5-data2.zip › Figure 5-source data 2/Raw Western blot for Figure 5E_â└-actin_unedited.tif]

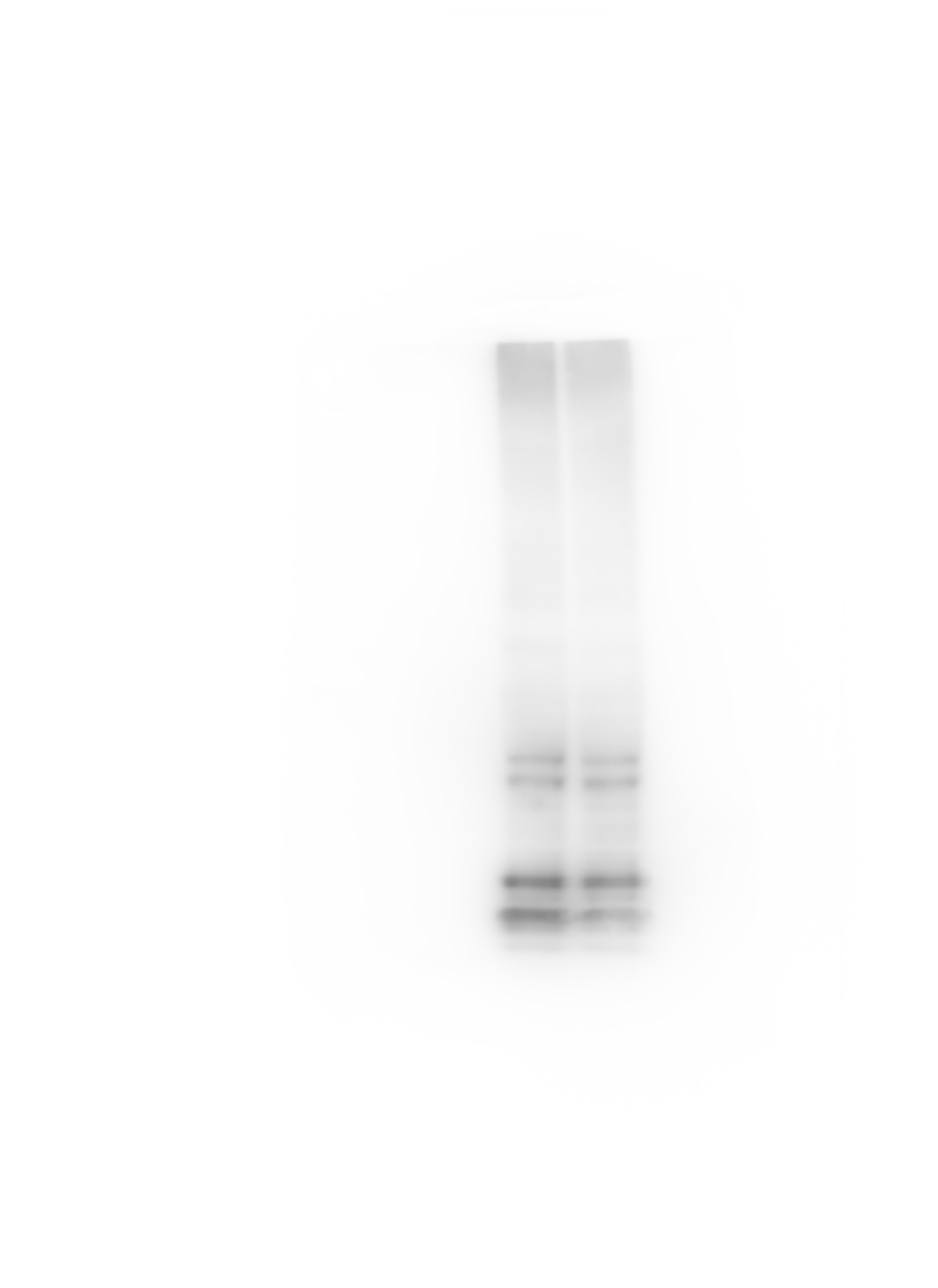

Supplement: Figure 5—source data 2. [file elife-92794-fig5-data2.zip › Figure 5-source data 2/Raw Western blot for Figure 5F_Input_FLAG_unedited.tif]

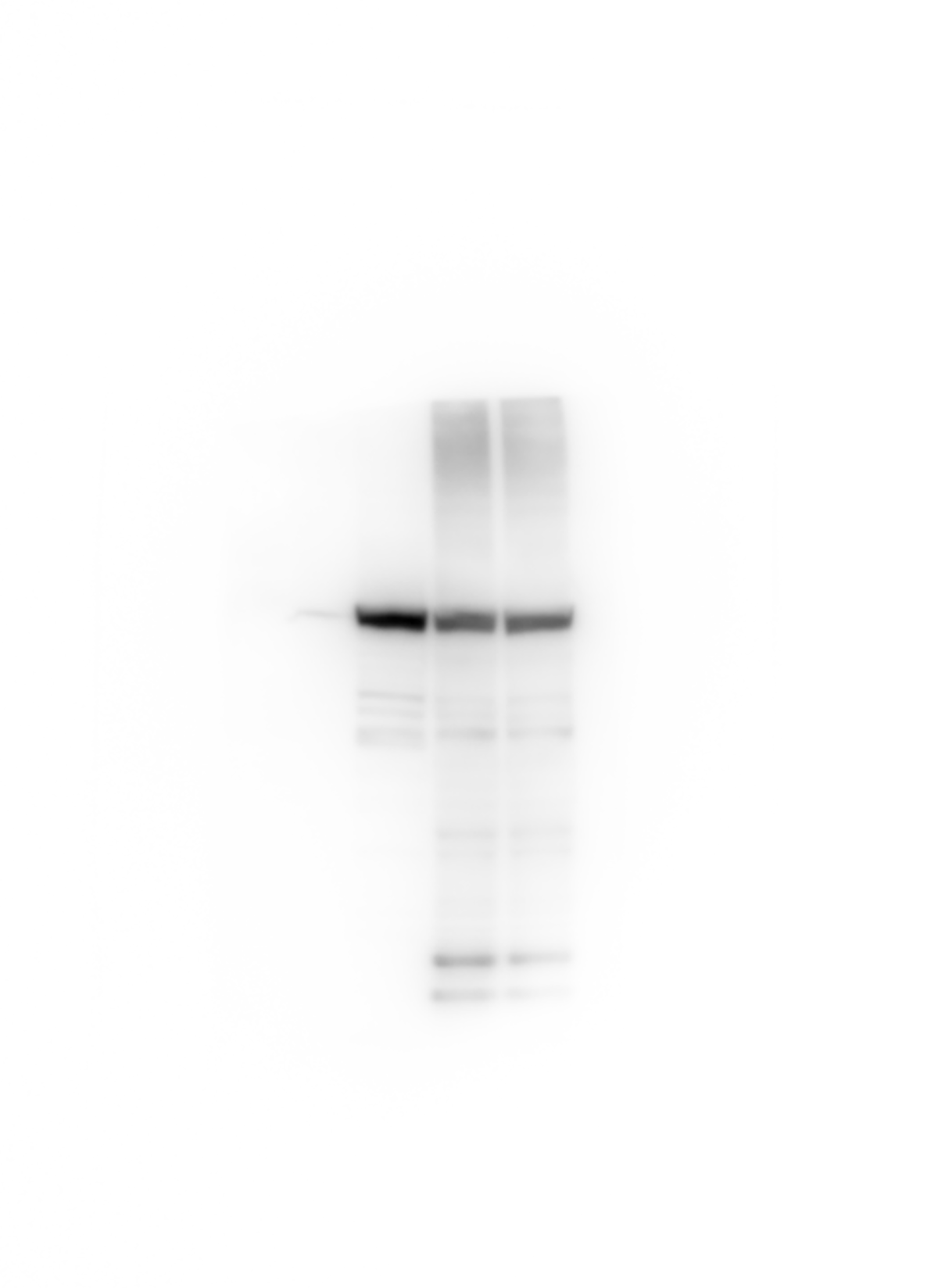

Supplement: Figure 5—source data 2. [file elife-92794-fig5-data2.zip › Figure 5-source data 2/Raw Western blot for Figure 5F_Input_PA_unedited.tif]

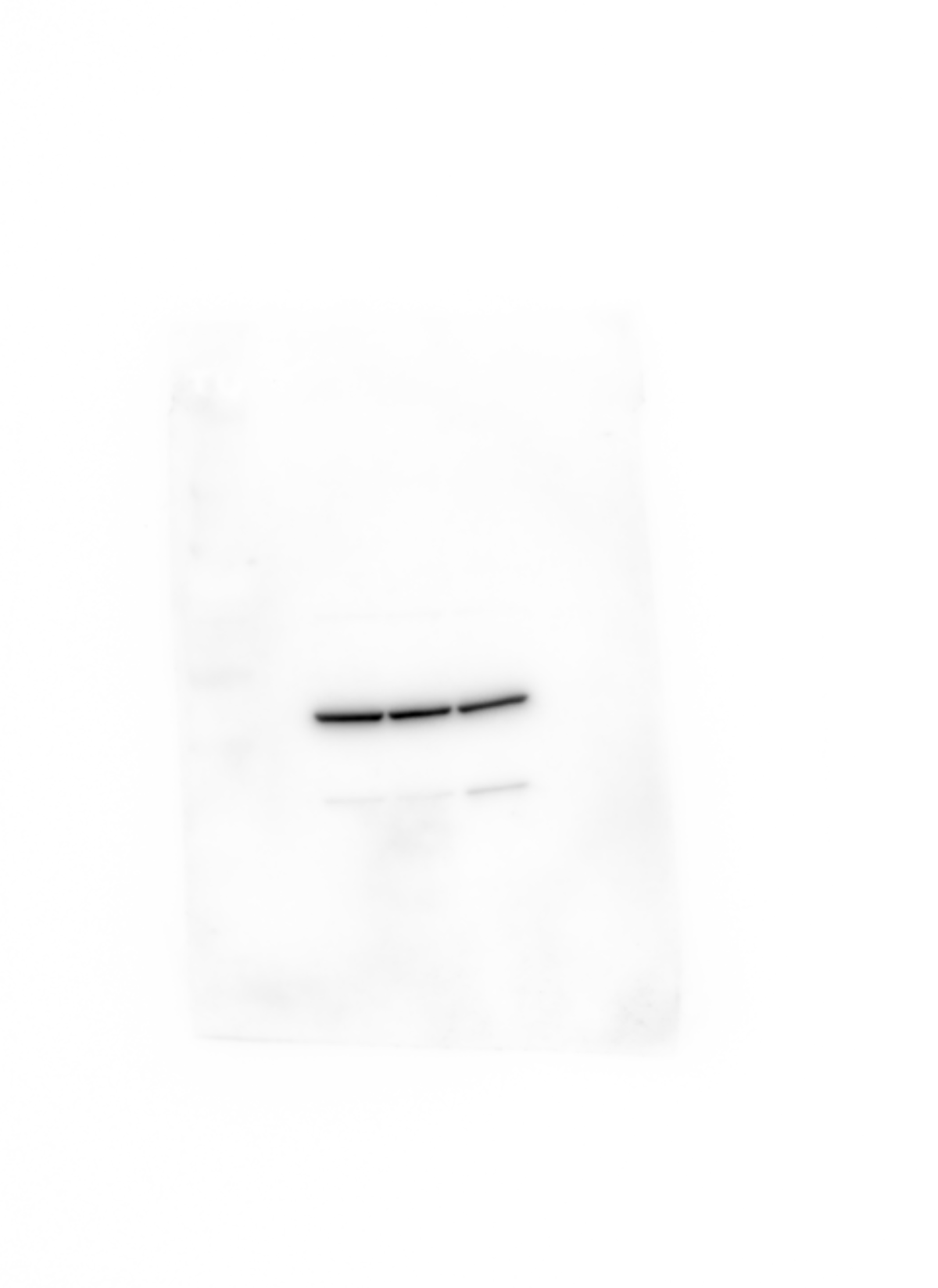

Supplement: Figure 5—source data 2. [file elife-92794-fig5-data2.zip › Figure 5-source data 2/Raw Western blot for Figure 5F_Input_â└-actin_unedited.tif]

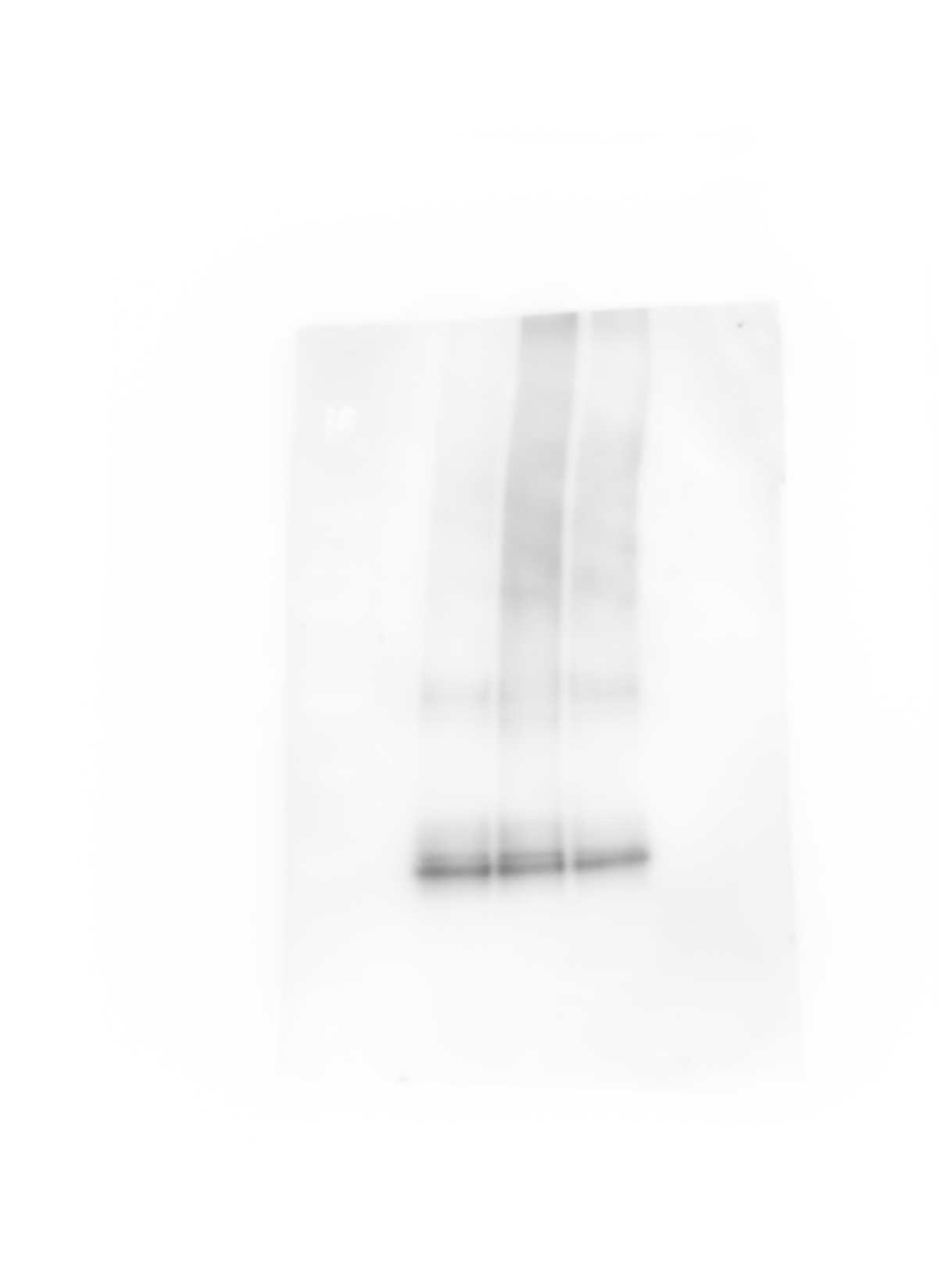

Supplement: Figure 5—source data 2. [file elife-92794-fig5-data2.zip › Figure 5-source data 2/Raw Western blot for Figure 5F_IP_HA_unedited.tif]

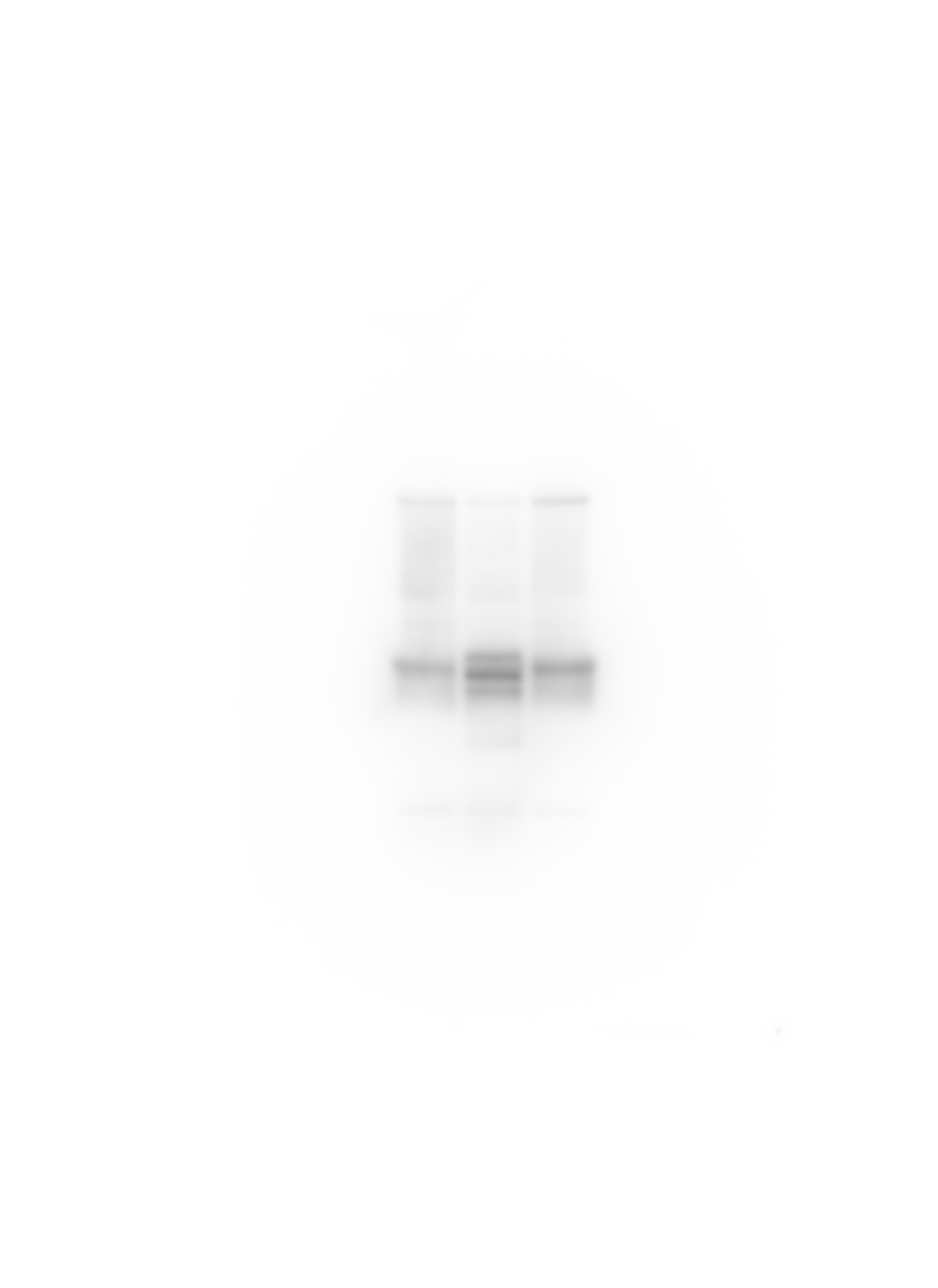

Supplement: Figure 5—source data 2. [file elife-92794-fig5-data2.zip › Figure 5-source data 2/Raw Western blot for Figure 5F_IP_PA_unedited.tif]

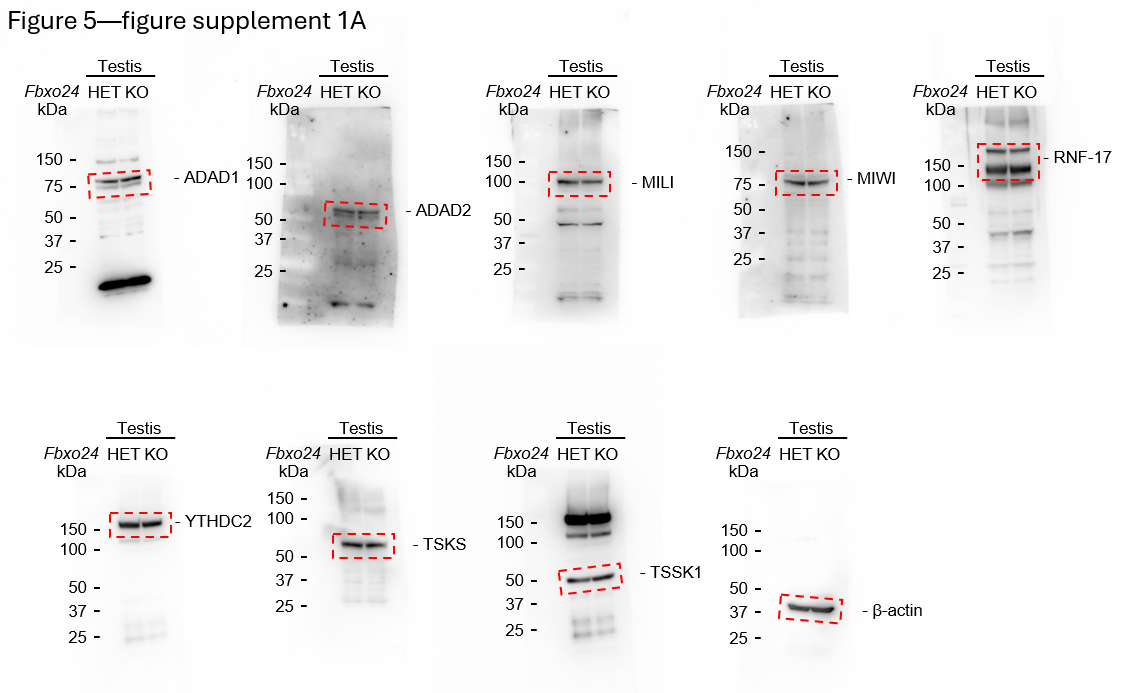

Supplement: Figure 5—figure supplement 1—source data 1. [file elife-92794-fig5-figsupp1-data1.zip › Figure 5-figure supplement 1-source data 1/Figure 5-figure supplement 1A_edited.tif]

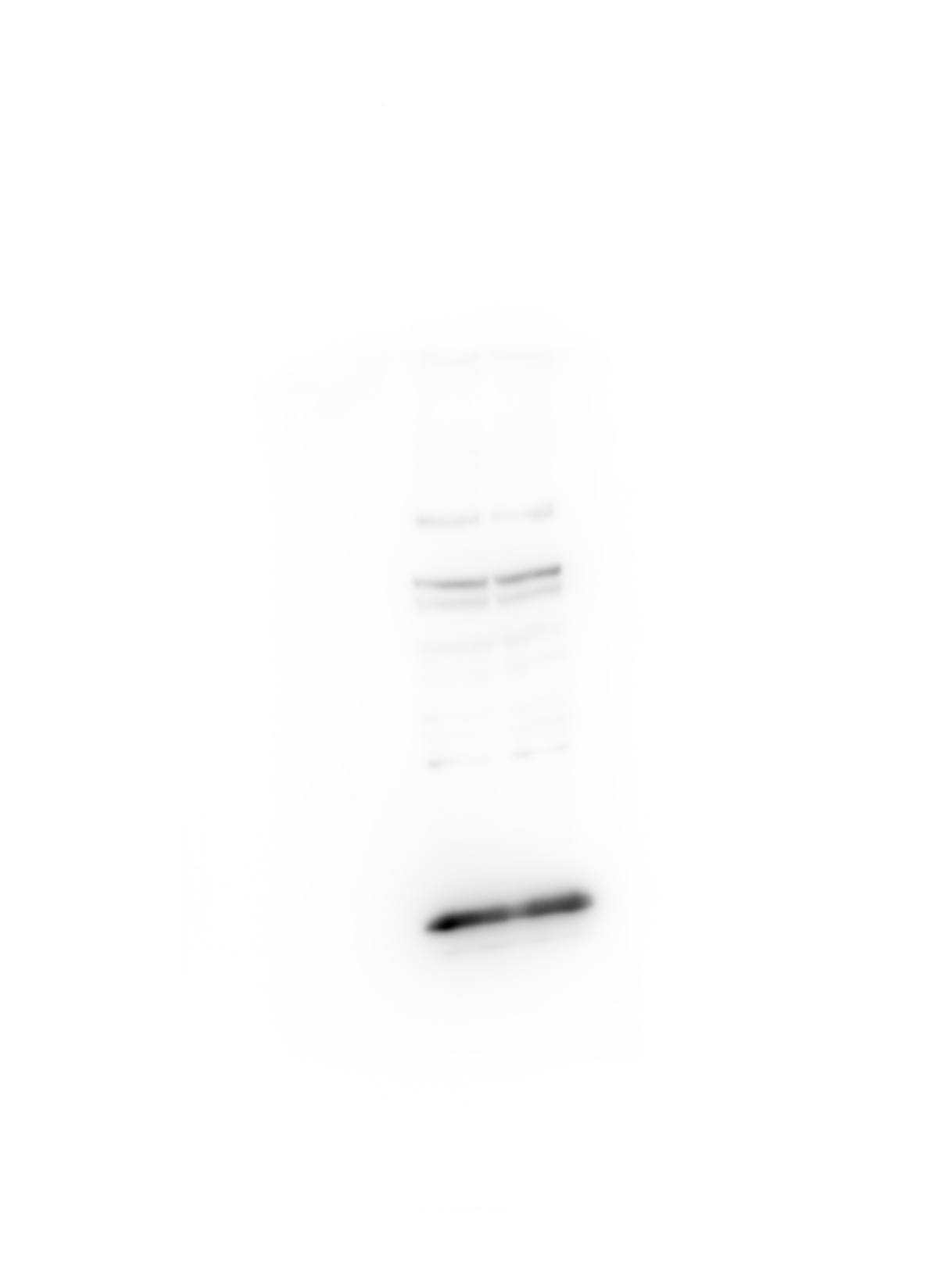

Supplement: Figure 5—figure supplement 1—source data 2. [file elife-92794-fig5-figsupp1-data2.zip › Figure 5-figure supplement 1-source data 2/Raw Western blot for Figure 5-figure supplement 1A_ADAD1_unedited.tif]

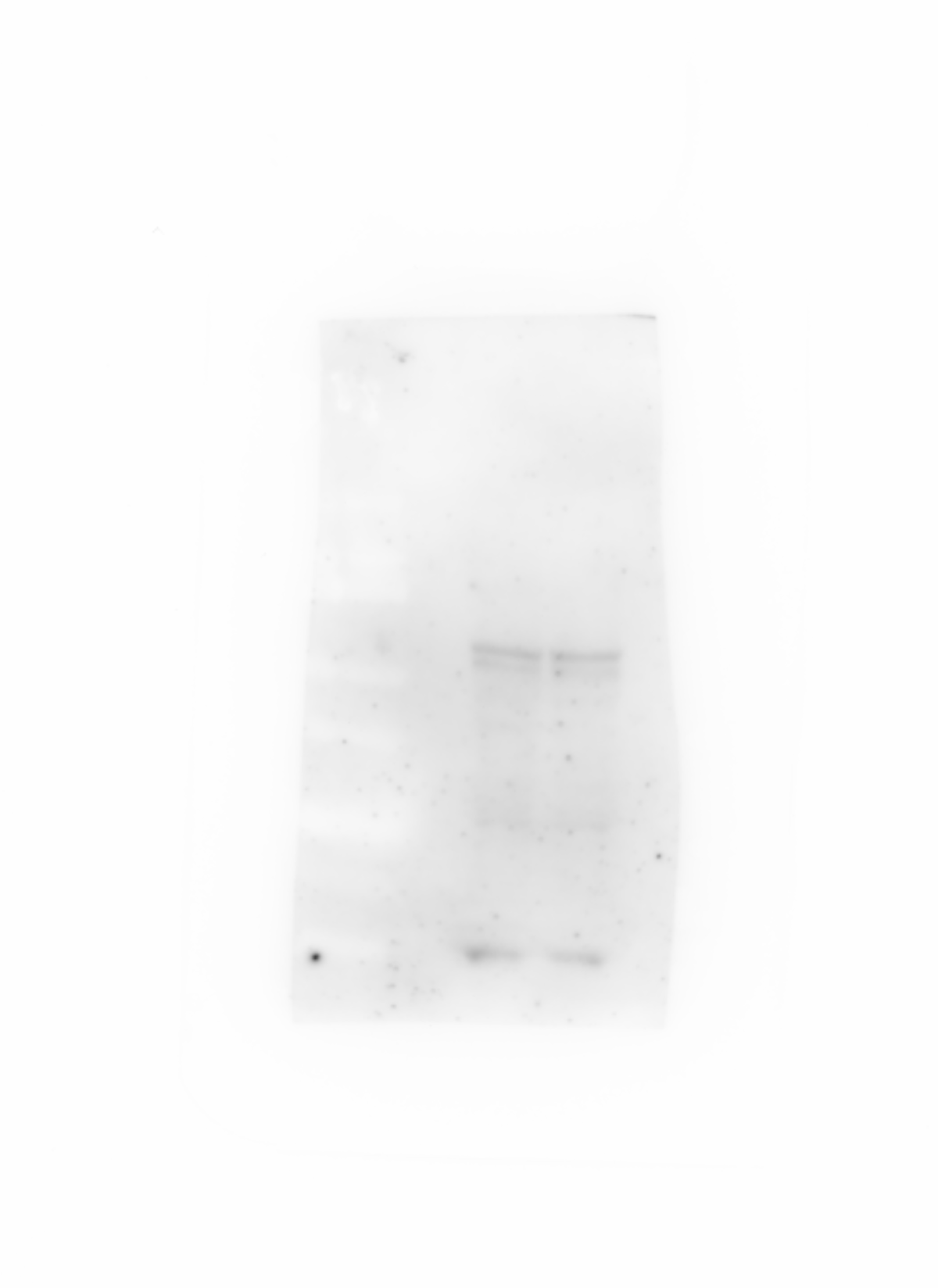

Supplement: Figure 5—figure supplement 1—source data 2. [file elife-92794-fig5-figsupp1-data2.zip › Figure 5-figure supplement 1-source data 2/Raw Western blot for Figure 5-figure supplement 1A_ADAD2_unedited.tif]

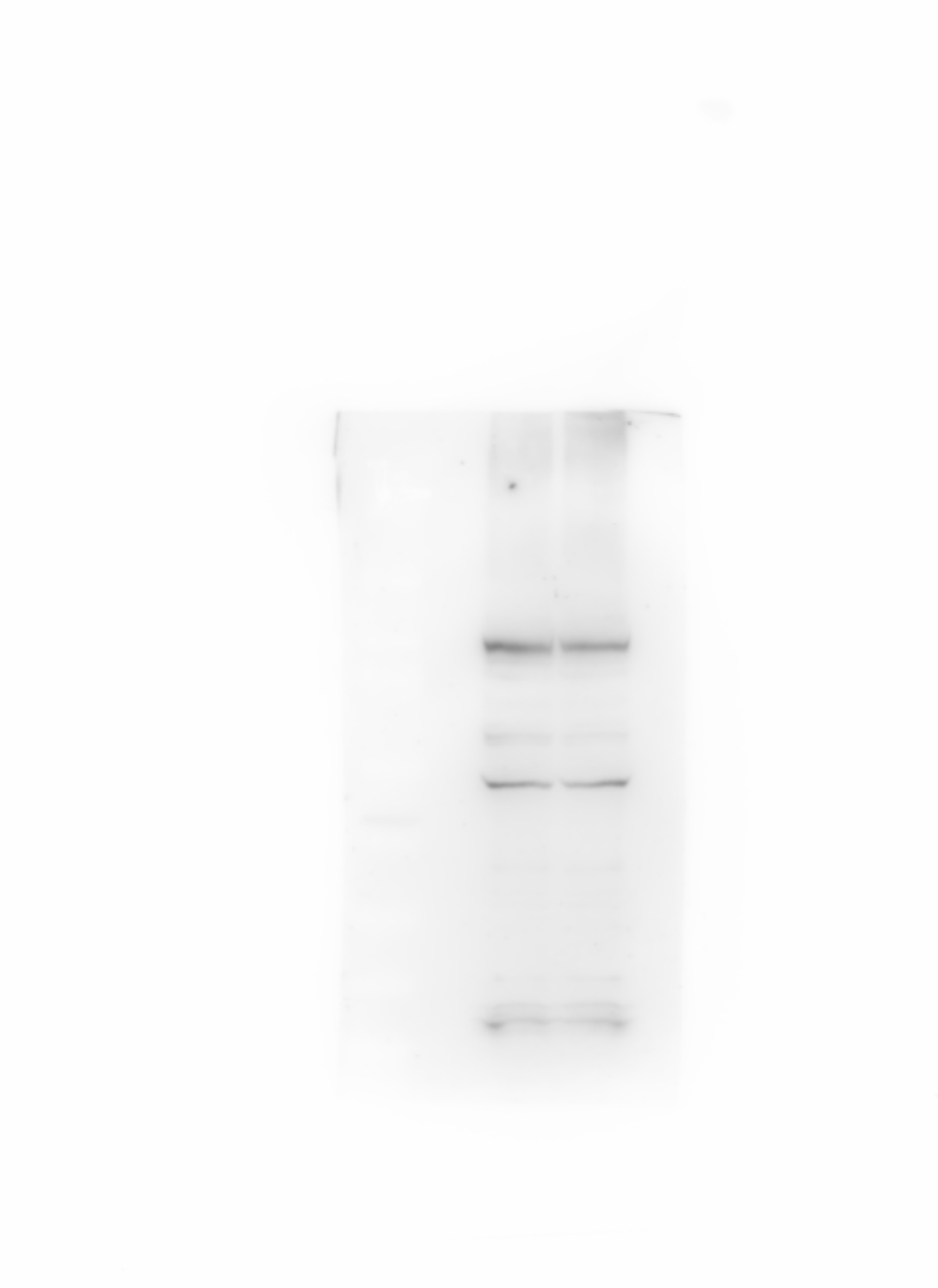

Supplement: Figure 5—figure supplement 1—source data 2. [file elife-92794-fig5-figsupp1-data2.zip › Figure 5-figure supplement 1-source data 2/Raw Western blot for Figure 5-figure supplement 1A_MILI_unedited.tif]

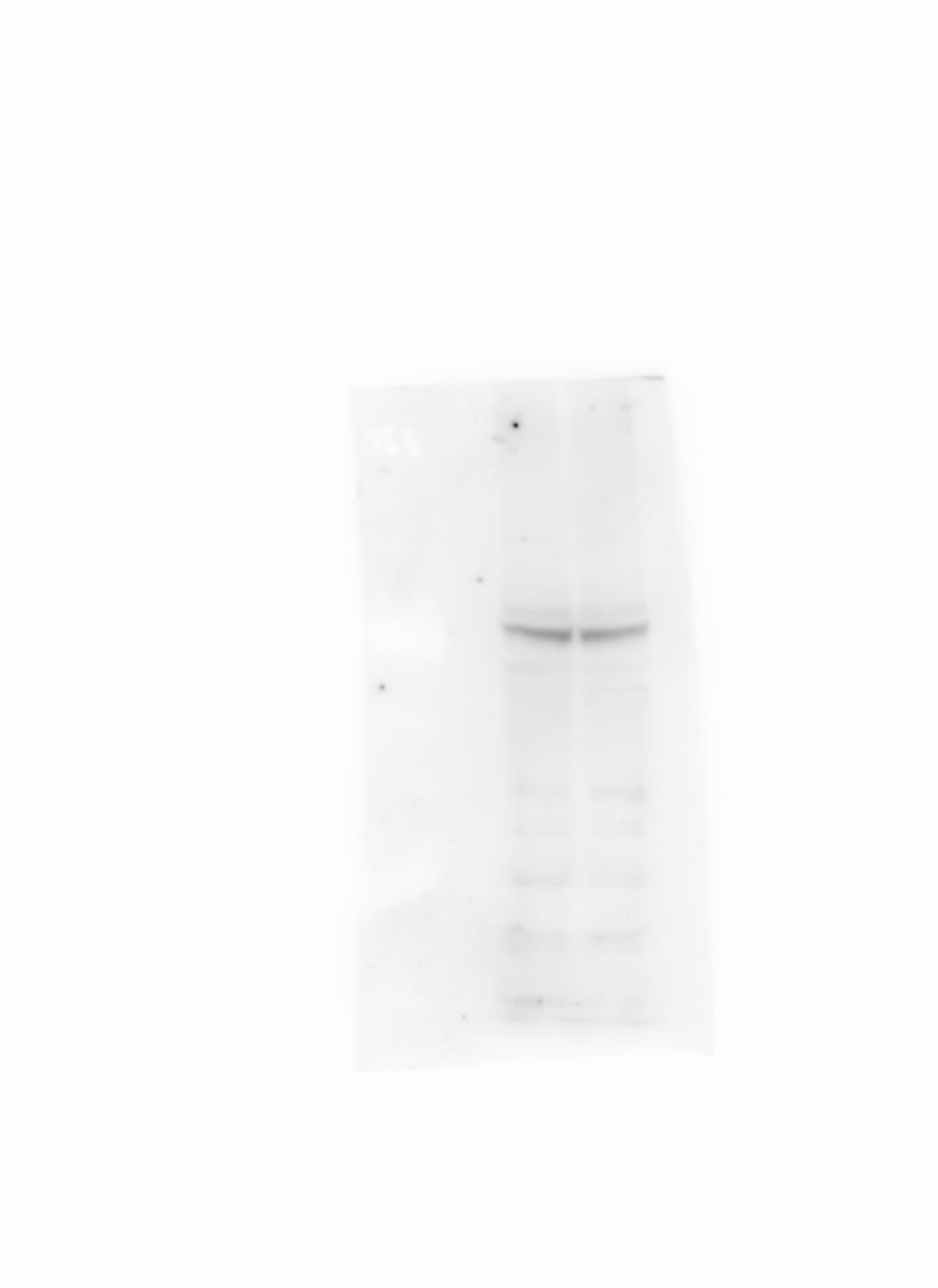

Supplement: Figure 5—figure supplement 1—source data 2. [file elife-92794-fig5-figsupp1-data2.zip › Figure 5-figure supplement 1-source data 2/Raw Western blot for Figure 5-figure supplement 1A_MIWI_unedited.tif]

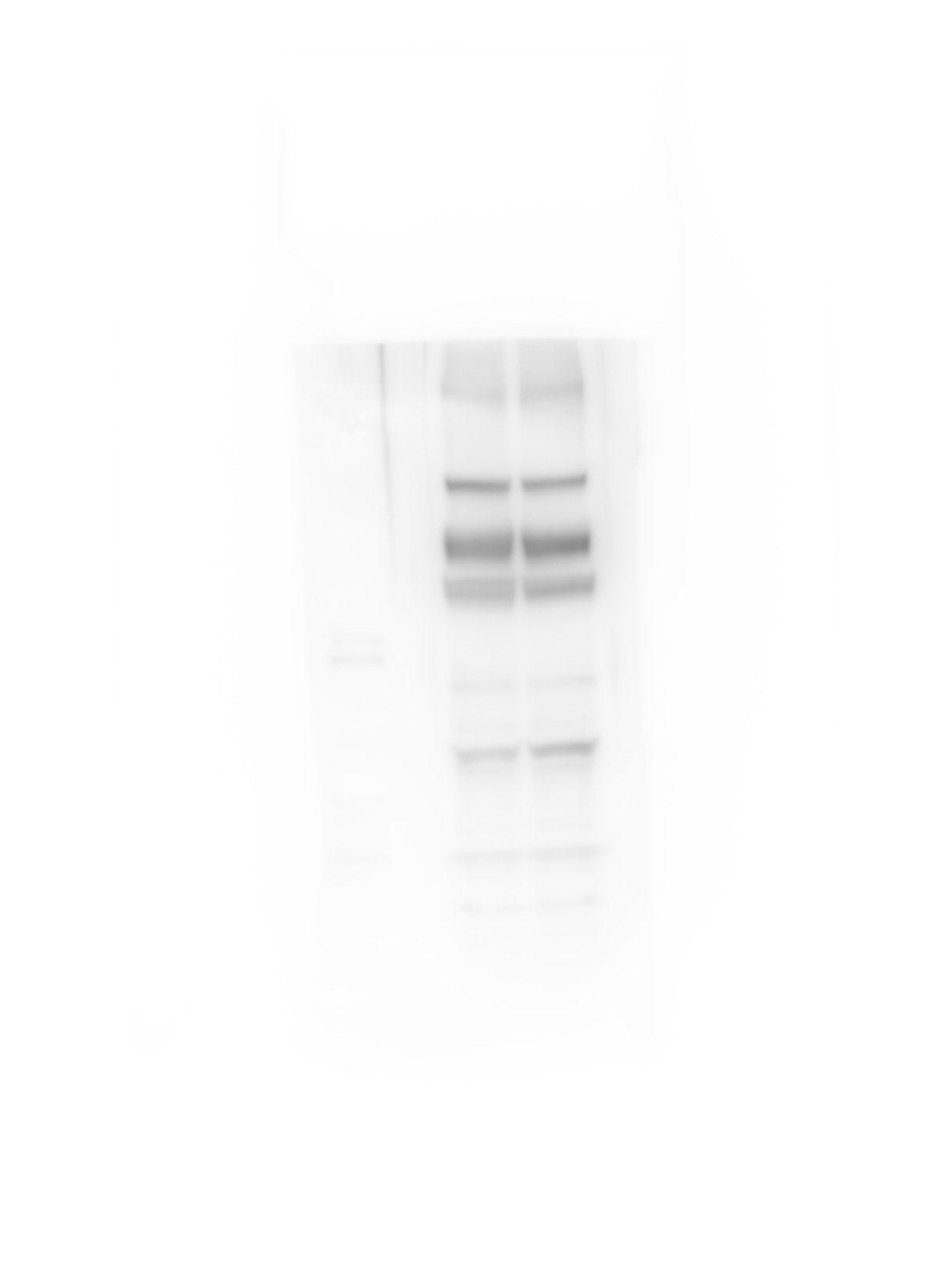

Supplement: Figure 5—figure supplement 1—source data 2. [file elife-92794-fig5-figsupp1-data2.zip › Figure 5-figure supplement 1-source data 2/Raw Western blot for Figure 5-figure supplement 1A_RNF-17_unedited.tif]

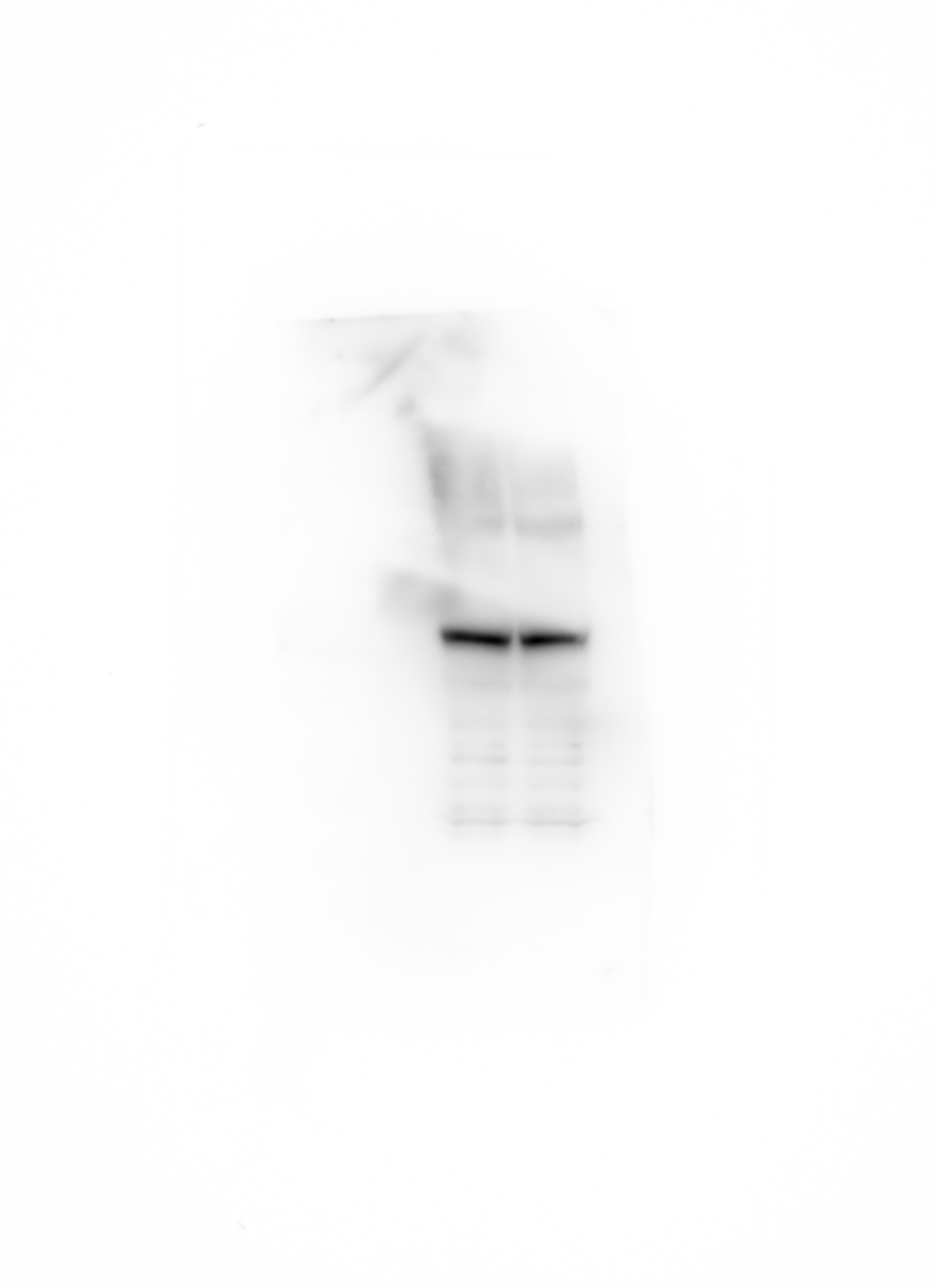

Supplement: Figure 5—figure supplement 1—source data 2. [file elife-92794-fig5-figsupp1-data2.zip › Figure 5-figure supplement 1-source data 2/Raw Western blot for Figure 5-figure supplement 1A_TSKS_unedited.tif]

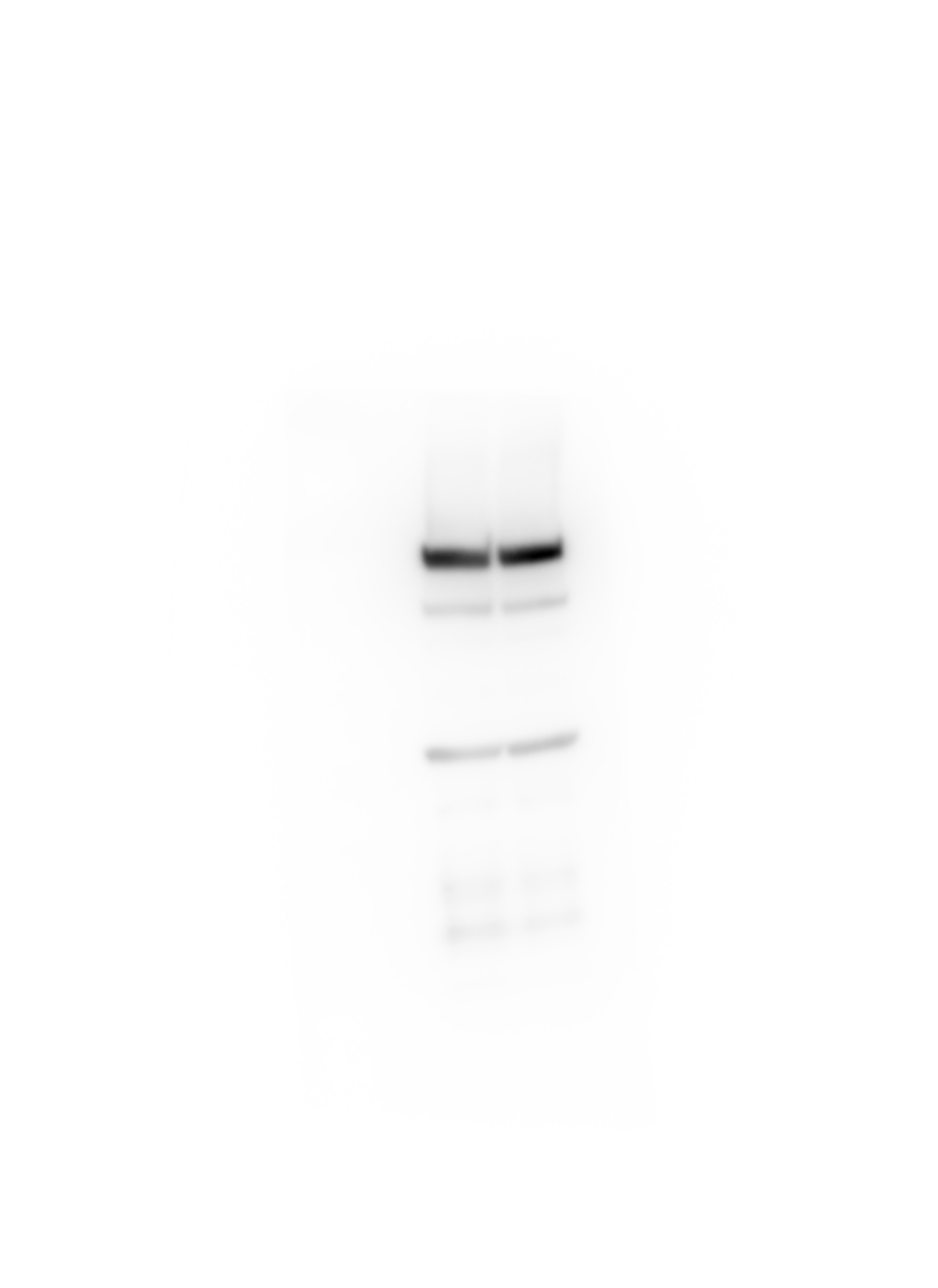

Supplement: Figure 5—figure supplement 1—source data 2. [file elife-92794-fig5-figsupp1-data2.zip › Figure 5-figure supplement 1-source data 2/Raw Western blot for Figure 5-figure supplement 1A_TSSK1_unedited.tif]

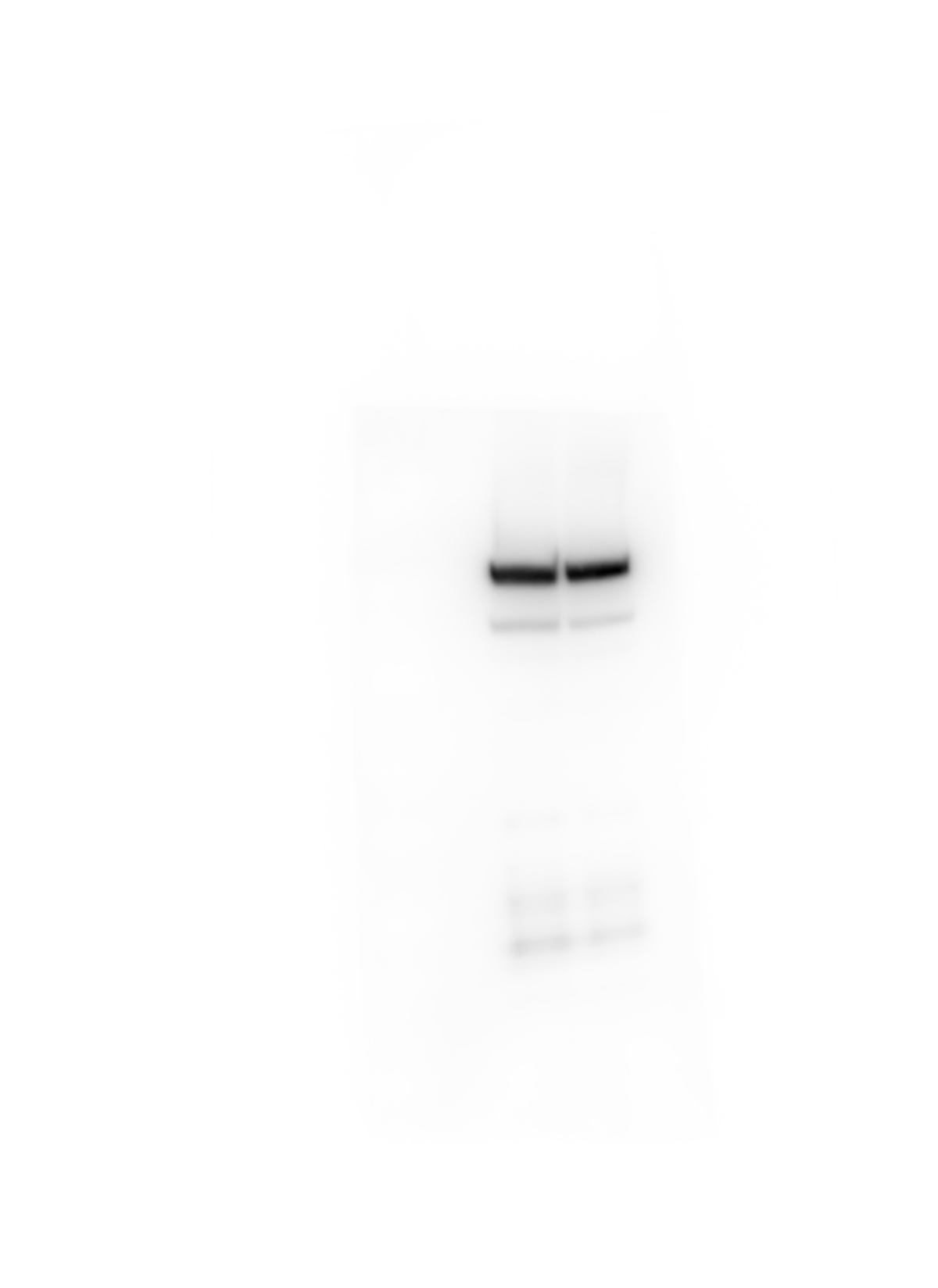

Supplement: Figure 5—figure supplement 1—source data 2. [file elife-92794-fig5-figsupp1-data2.zip › Figure 5-figure supplement 1-source data 2/Raw Western blot for Figure 5-figure supplement 1A_YTHDC2_unedited.tif]

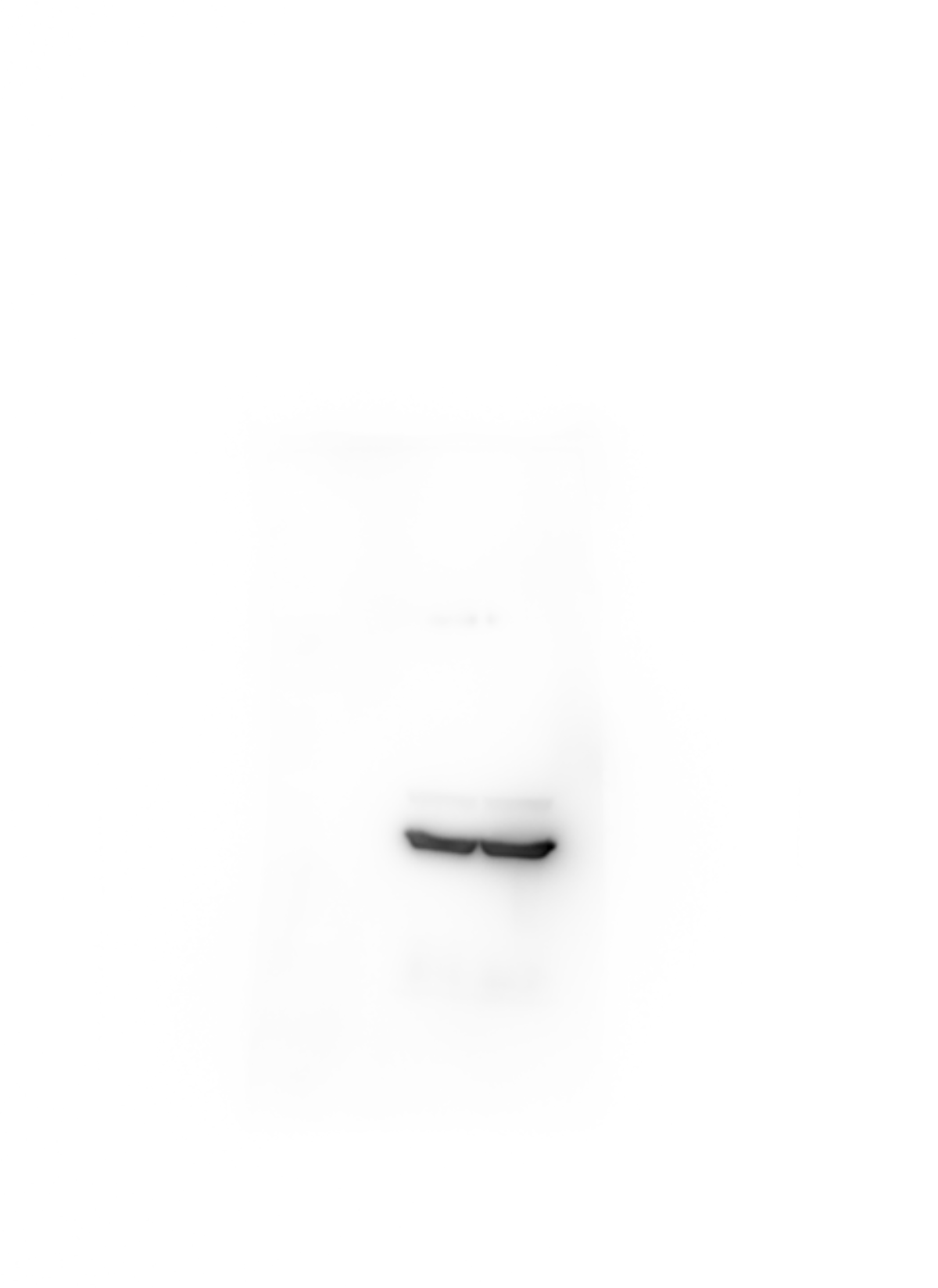

Supplement: Figure 5—figure supplement 1—source data 2. [file elife-92794-fig5-figsupp1-data2.zip › Figure 5-figure supplement 1-source data 2/Raw Western blot for Figure 5-figure supplement 1A_â└-actin_unedited.tif]

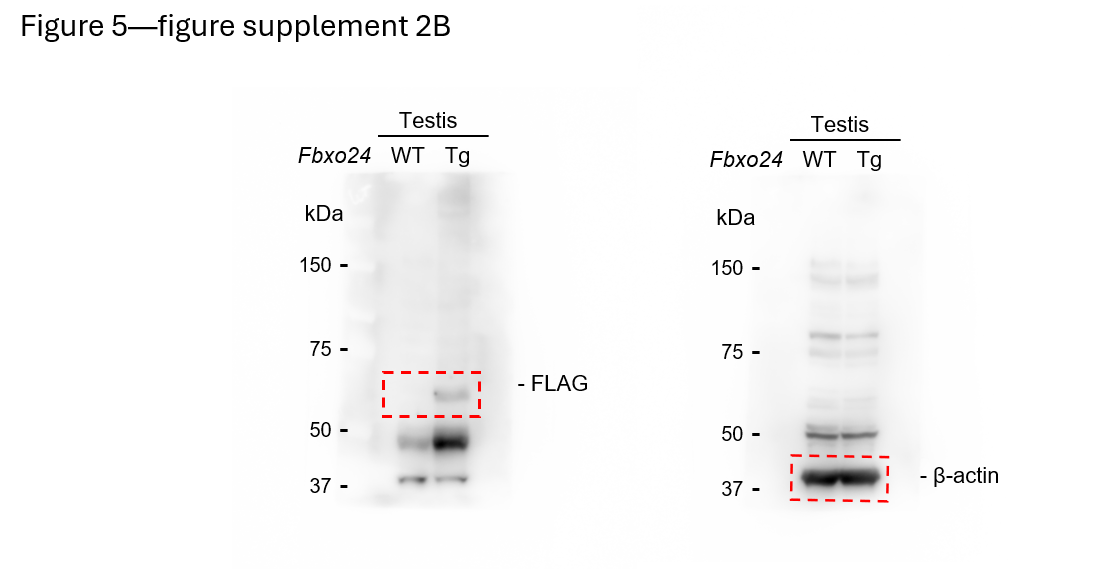

Supplement: Figure 5—figure supplement 2—source data 1. [file elife-92794-fig5-figsupp2-data1.zip › Figure 5-figure supplement 2-source data 1/Figure 5-figure supplement 2B_edited.tif]

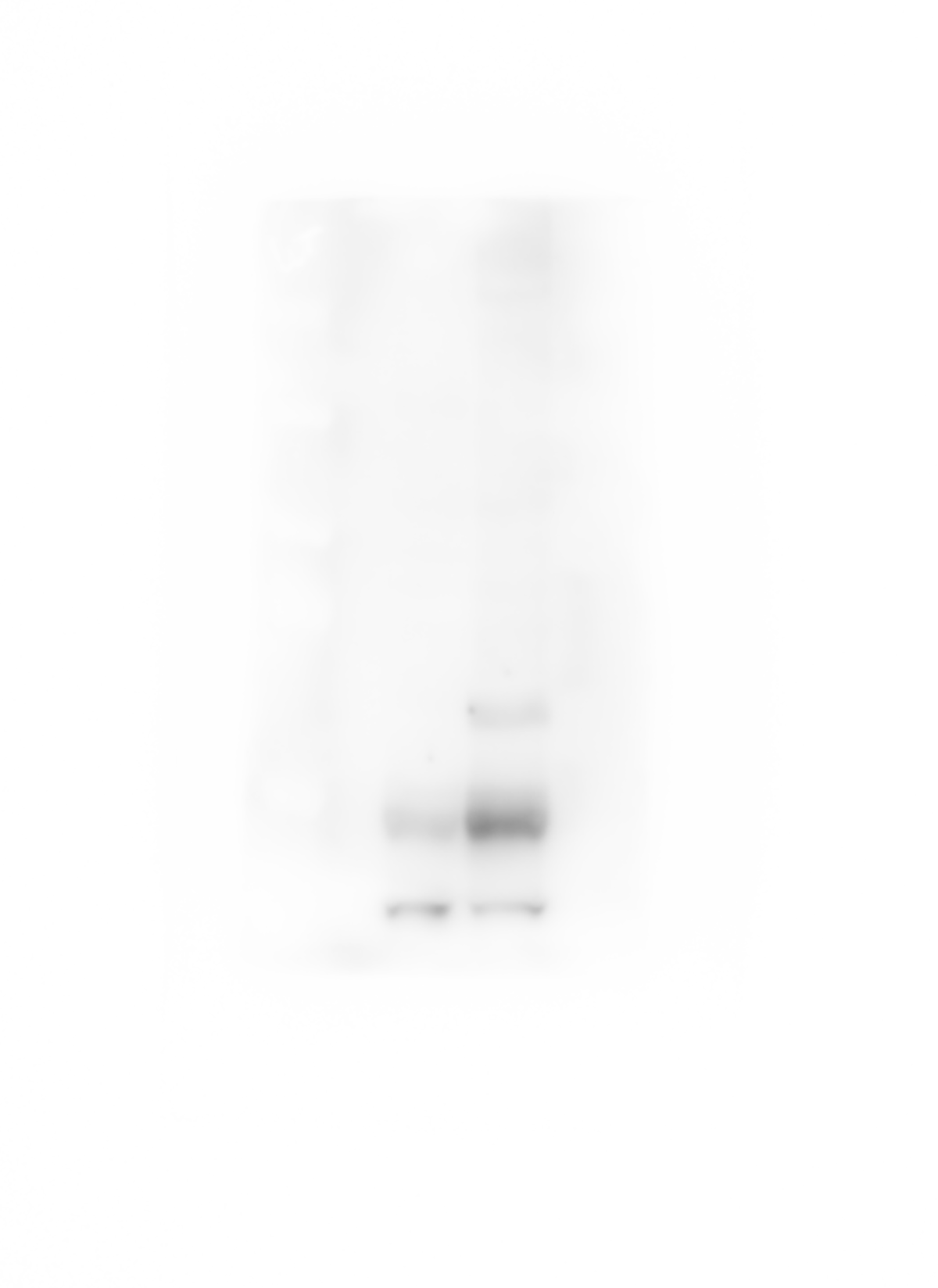

Supplement: Figure 5—figure supplement 2—source data 2. [file elife-92794-fig5-figsupp2-data2.zip › Figure 5-figure supplement 2-source data 2/Raw western blot for Figure 5-figure supplement 2B_FLAG_unedited.tif]

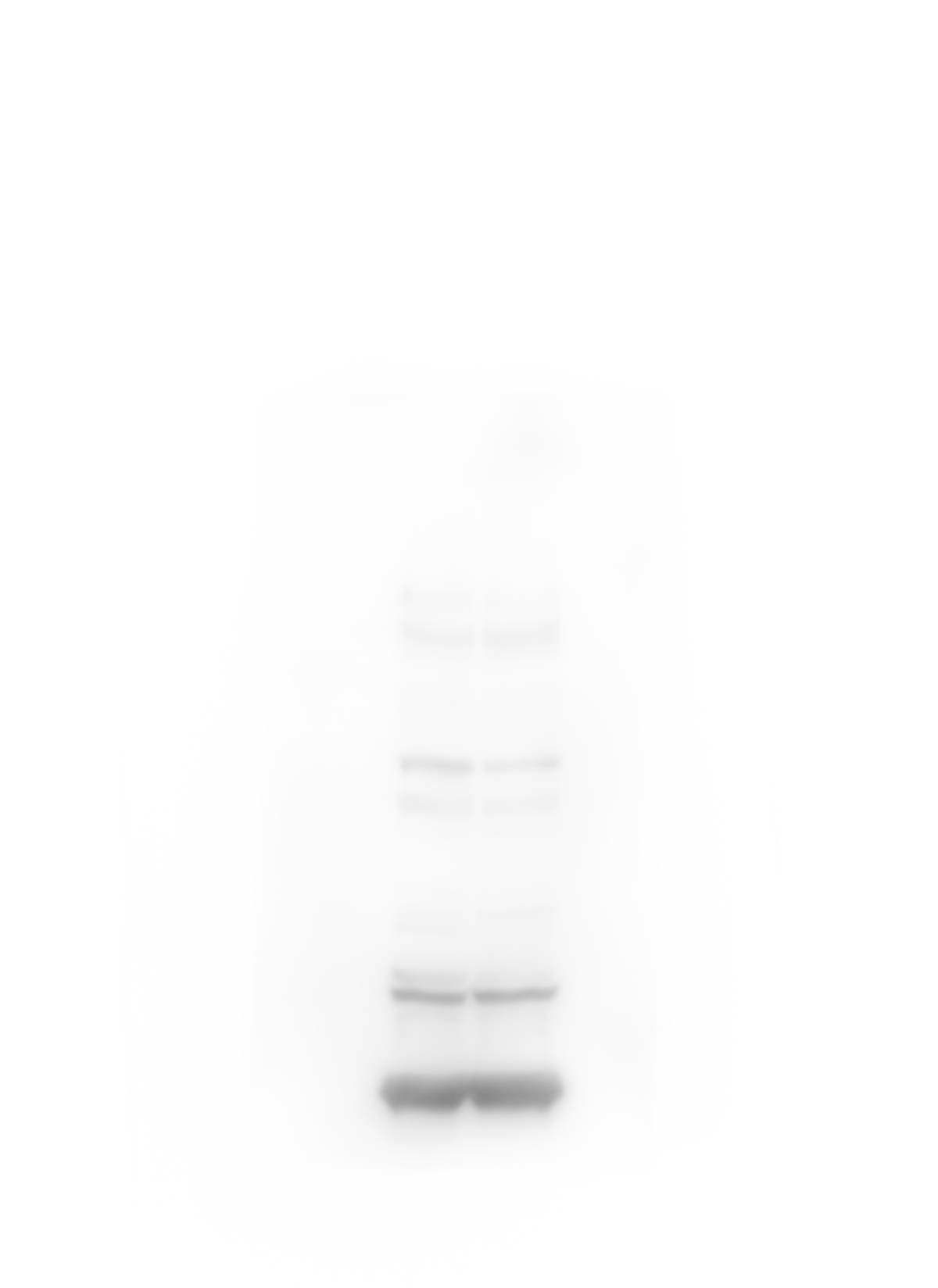

Supplement: Figure 5—figure supplement 2—source data 2. [file elife-92794-fig5-figsupp2-data2.zip › Figure 5-figure supplement 2-source data 2/Raw western blot for Figure 5-figure supplement 2B_â└-actin_unedited.tif]

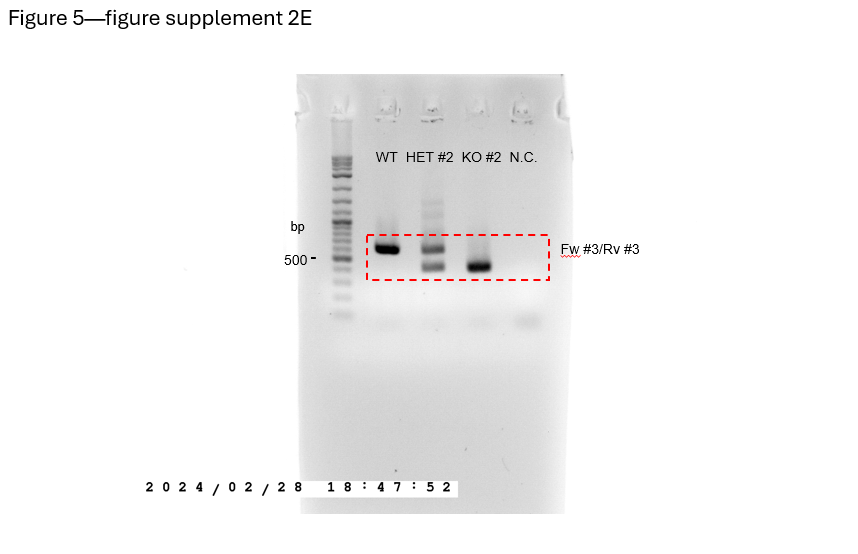

Supplement: Figure 5—figure supplement 2—source data 3. [file elife-92794-fig5-figsupp2-data3.zip › Figure 5-figure supplement 2-source data 3/Figure 5-figure supplement 2E_edited.tif]

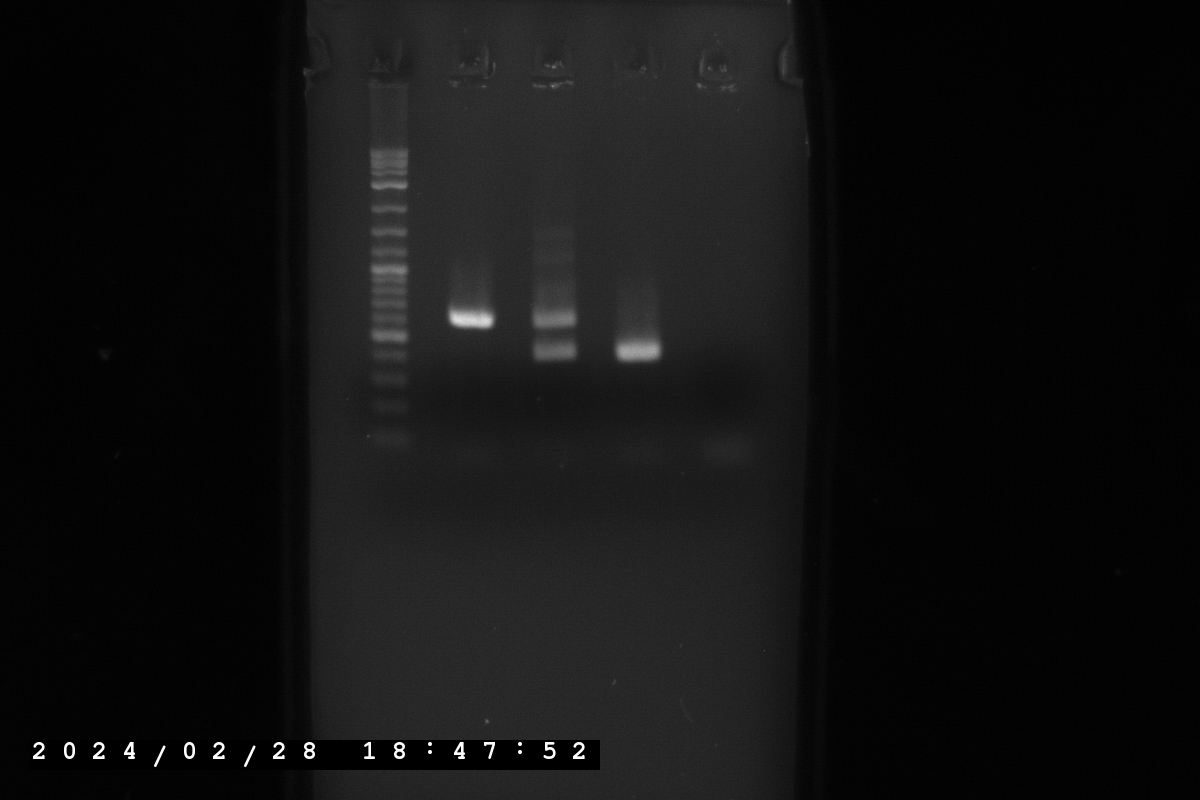

Supplement: Figure 5—figure supplement 2—source data 4. [file elife-92794-fig5-figsupp2-data4.zip › Figure 5-figure supplement 2-source data 4/Raw genotyping gel for Figure 5-figure supplement 2E_unedited.tiff]

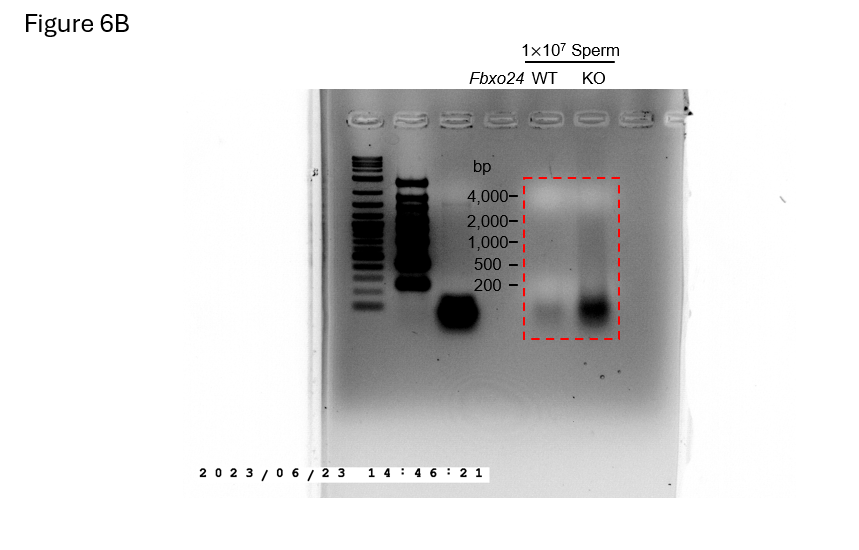

Supplement: Figure 6—source data 1. [file elife-92794-fig6-data1.zip › Figure 6-source data 1/Figure 6B_edited.tif]

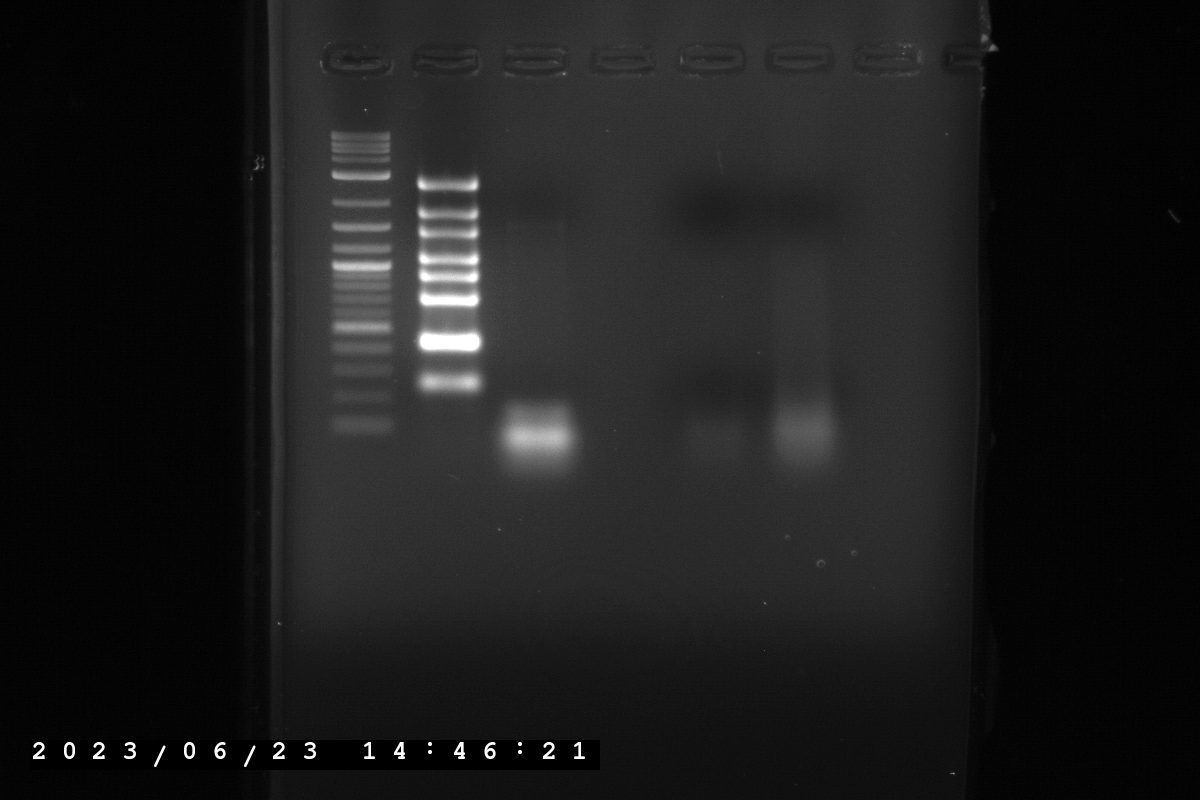

Supplement: Figure 6—source data 2. [file elife-92794-fig6-data2.zip › Figure 6-source data 2/Raw RNA electrophoresis gel for Figure 6B.tiff]
